# Skeletal muscle mitochondrial health in type 1 diabetes: the role of exercise capacity and lifestyle factors

**DOI:** 10.1007/s00125-025-06451-1

**Published:** 2025-05-21

**Authors:** Richie P. Goulding, Braeden T. Charlton, Ellen A. Breedveld, Jelle Y. Huijts, Matthijs van der Laan, Anne R. Strating, Wendy Noort, Aryna Kolodyazhna, Anita E. Grootemaat, Frank W. Bloemers, Nicole N. van der Wel, Rob C. I. Wüst

**Affiliations:** 1 Department of Human Movement Sciences, Faculty of Behavioural and Movement Sciences, Vrije Universiteit Amsterdam, Amsterdam Movement Sciences, Amsterdam, the Netherlands; 2 Electron Microscopy Centre Amsterdam, Amsterdam UMC, Location Academic Medical Centre, Amsterdam, the Netherlands; 3 Department of Trauma Surgery, Amsterdam Movement Sciences, Amsterdam UMC, Vrije Universiteit Amsterdam, Amsterdam, the Netherlands

**Keywords:** Maximal oxygen uptake, Mitochondrial density, Mitochondrial respiration, Muscle bioenergetics, Muscle mitochondria, Skeletal muscle, Type 1 diabetes

## Abstract

**Aims/hypothesis:**

Previous studies reporting lower skeletal muscle mitochondrial function in type 1 diabetes did not account for cardiorespiratory fitness, a key confounder when assessing mitochondrial function. We hypothesised that, compared with healthy individuals, muscle mitochondrial phenotypic differences would be abolished in individuals with type 1 diabetes when matched for age, sex, BMI and maximal oxygen uptake ($\dot{V}{\text{O}}_{\text{2max}}$).

**Methods:**

Seventeen individuals with type 1 diabetes and seventeen healthy control individuals matched for age, sex, BMI and $\dot{V}{\text{O}}_{\text{2max}}$ participated and underwent a muscle biopsy from the vastus lateralis. Mitochondrial respiration was assessed by high-resolution respirometry, and mitochondrial density and morphology were assessed by transmission electron microscopy.

**Results:**

$\dot{V}{\text{O}}_{\text{2max}}$ (individuals with type 1 diabetes 40±10 kg^−1^ min^−1^; control individuals 41±8 ml kg^−1^ min^−1^; *p*=0.51) and mitochondrial oxidative phosphorylation capacity (individuals with type 1 diabetes 101±35 [pmol O_2_] s^−1^ mg^−1^; control individuals 99±23 [pmol O_2_] s^−1^ mg^−1^, *p*=0.82) did not differ between groups. Both intermyofibrillar (individuals with type 1 diabetes 6.07±2.16%; control individuals 6.01±1.11%; *p*=0.92) and subsarcolemmal (individuals with type 1 diabetes 18.70±8.16%; control individuals 19.29±7.36%; *p*=0.83) mitochondrial densities were not different between groups. Mitochondrial respiration normalised by density did not differ between groups. However, individuals with type 1 diabetes and higher HbA_1c_ displayed lower rates of mitochondrial respiration than those with lower HbA_1c_, whereas those with higher BMI displayed lower mitochondrial densities than those with lower BMI.

**Conclusions/interpretation:**

Collectively, our study demonstrates that when matched for age, sex, BMI and $\dot{V}{\text{O}}_{\text{2max}}$, maximal muscle mitochondrial respiration and morphology in people with type 1 diabetes are not impaired. These findings highlight the importance of habitual exercise, optimal glucose management and a healthy BMI in maintaining mitochondrial health in individuals with type 1 diabetes.

**Graphical Abstract:**

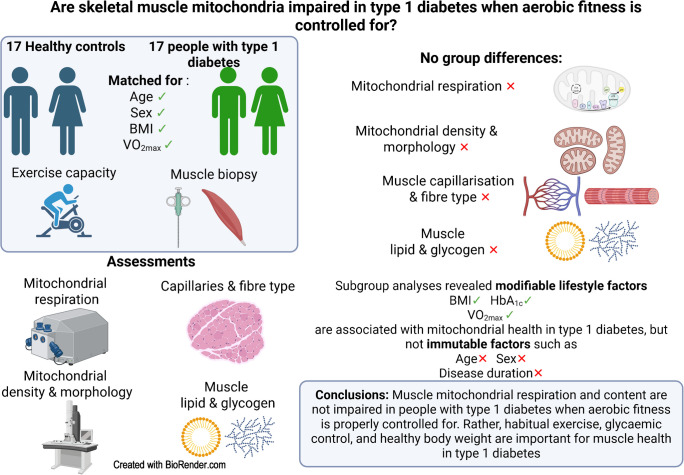

**Supplementary Information:**

The online version of this article (10.1007/s00125-025-06451-1) contains peer-reviewed but unedited supplementary material.


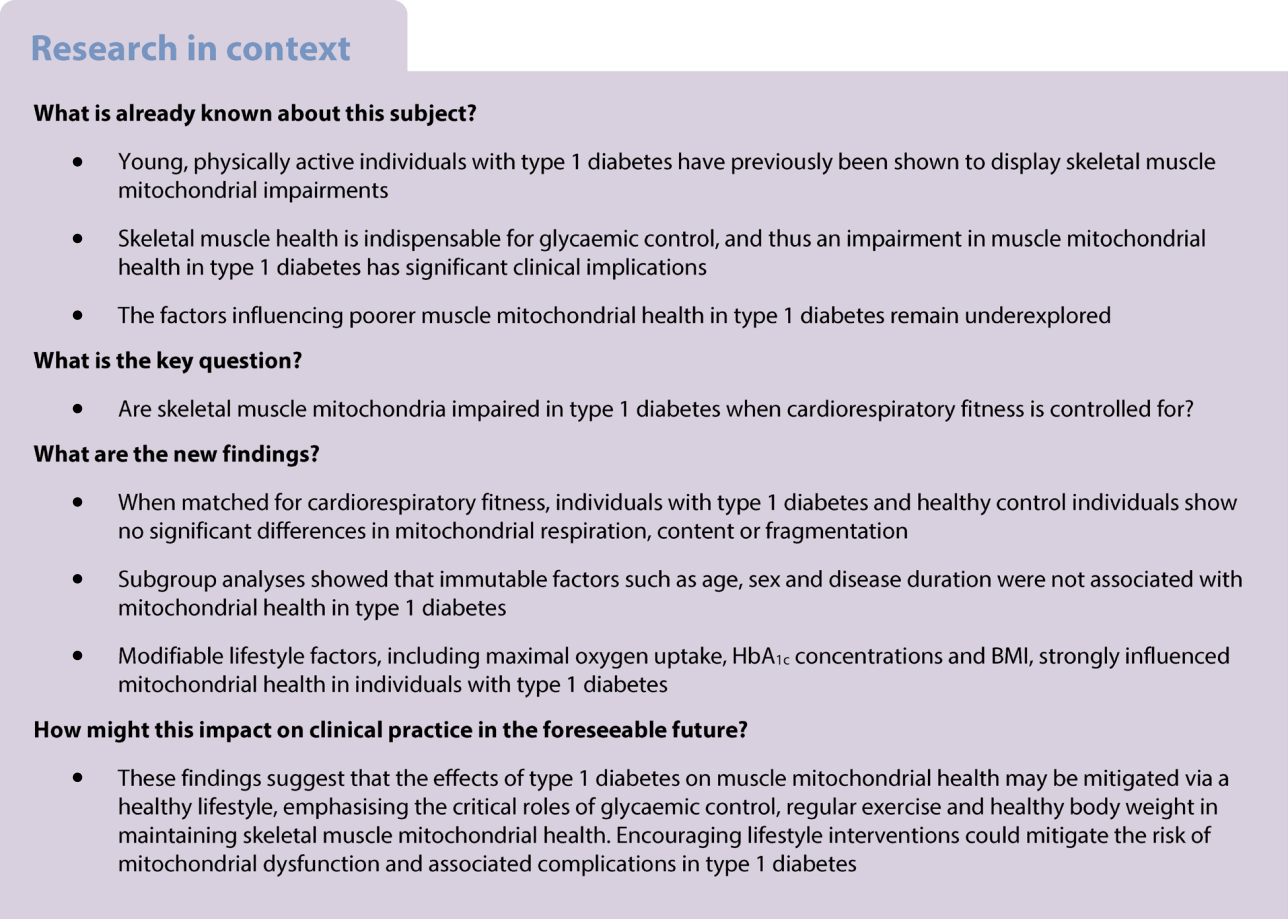


## Introduction

Type 1 diabetes is an autoimmune disease resulting in destruction of pancreatic beta cells. The resulting insulin deficiency causes an inability to maintain glucose homeostasis and reliance on the administration of exogenous insulin. This peripherally administered insulin bypasses the canonical liver-first insulin delivery via the portal vein [1], shifting the emphasis of insulin’s action towards peripheral tissues, of which skeletal muscle is the largest and most metabolically active. Hence, skeletal muscle is the primary organ involved in glucose homeostasis in type 1 diabetes.

As skeletal muscle mitochondria are primarily responsible for metabolising glucose, impaired mitochondrial health in type 1 diabetes would impede the ability to maintain glucose homeostasis and accelerate the development of diabetic complications [2, 3]. Impaired in vivo [4–6] and ex vivo [7–9] skeletal muscle mitochondrial respiration have been reported in individuals with type 1 diabetes compared with healthy control individuals. In addition, abnormal mitochondrial cristae morphology [8] and reduced muscle mitochondrial content [9] were observed in individuals with type 1 diabetes compared with healthy control individuals. However, these findings are not universal, with some studies reporting no differences in in vivo [10] or ex vivo [11] mitochondrial respiration between those with type 1 diabetes and healthy individuals, and others reporting enhanced ex vivo intrinsic mitochondrial respiratory capacity in individuals with type 1 diabetes [9]. A notable limitation of these studies is the lack of control for whole-body cardiorespiratory fitness (i.e. maximal oxygen uptake [$\dot{V}{\text{O}}_{\text{2max}}$]). Because mitochondrial content and oxidative capacity are so closely related to $\dot{V}{\text{O}}_{\text{2max}}$ [12], it remains unclear whether the observed mitochondrial impairments are intrinsic to type 1 diabetes per se, or secondary to lower aerobic fitness. $\dot{V}{\text{O}}_{\text{2max}}$ is typically reduced in type 1 diabetes [13, 14], further complicating the interpretation of these findings.

Another issue complicating the understanding of how type 1 diabetes impacts mitochondria is the interaction between the disease and other physiological and metabolic predictors of mitochondrial health. Greater adiposity (reflected by greater BMI) may predispose individuals with type 1 diabetes towards compromised mitochondrial health given its link with insulin resistance [15]. Ageing is associated with diminished mitochondrial bioenergetic function [16, 17]; however, older individuals with type 1 diabetes have been demonstrated to display normal intrinsic mitochondrial respiratory capacity [9]. Disease duration may also be a factor, as prolonged exposure to the metabolic and systemic effects of type 1 diabetes could result in impaired mitochondrial health. It has been hypothesised that diabetes complications arise due to repeated bouts of hyperglycaemia [2, 3], thus suboptimal glycaemic control (reflected by greater HbA_1c_) could be associated with impaired mitochondrial health. However, how these physiological and metabolic predictors interact with type 1 diabetes to determine mitochondrial health remains unclear.

In the present study, we aimed to separate primary disease effects from those related to lifestyle factors known to interfere with mitochondrial health, such as reduced aerobic training status, suboptimal glycaemic control and high BMI. We examined skeletal muscle mitochondrial characteristics in individuals with type 1 diabetes and healthy control individuals matched for age, sex, BMI and $\dot{V}{\text{O}}_{\text{2max}}$. We hypothesised that individuals with type 1 diabetes would display similar mitochondrial characteristics to those of healthy control individuals. A secondary aim was to determine the impact of age, sex, disease duration, BMI and glycaemic control on mitochondrial characteristics in individuals with type 1 diabetes. Our hypothesis was that modifiable lifestyle factors such as BMI and HbA_1c_ would be significant predictors of mitochondrial health in type 1 diabetes.

## Methods

Further methodological details are given in electronic supplementary material (ESM) Methods, with a flowchart of participant screening and recruitment presented in ESM Fig. 1.

### Participants

Seventeen individuals with type 1 diabetes and 17 control individuals with similar age, sex, BMI, physical activity and $\dot{V}{\text{O}}_{\text{2max}}$ participated. The study was approved by the Amsterdam UMC Medical Ethics Committee (NL76008.029.20) and conformed to the Declaration of Helsinki. All participants provided informed consent prior to participation.

### Experimental overview

Each participant visited the laboratory twice. At the initial visit, participants completed an incremental ramp exercise test on a cycle ergometer (Lode Excalibur Sport, the Netherlands) for determination of $\dot{V}{\text{O}}_{\text{2max}}$. At the second visit, a muscle biopsy was taken from the vastus lateralis using a suction-supported 5 mm Bergström needle. Samples were stretched and frozen in liquid nitrogen for later analysis or stored as otherwise stated. Prior to the first visit, participants completed a 7 day physical activity diary. Prior to all visits, participants arrived 3 h postprandial, avoiding alcohol, caffeine and strenuous exercise in the previous 24 h. Prior to the biopsy, participants omitted their fast-acting insulin but maintained their basal insulin. HbA_1c_ was measured via capillary blood sample using a portable analyser (HbA_1c_Now^+^, PTS Diagnostics, USA).

### Exercise testing procedures

The exercise test began with 2 min of rest, followed by 4 min baseline cycling at 30–40 W, followed by a ramped increase in power (20–30 W/min, depending on sex, body mass and anticipated fitness). Cadence was maintained between 70–90 rev/min; task failure was defined as cadence dropping below 60 rev/min despite verbal encouragement. Pulmonary gas exchange and ventilation were measured breath-by-breath (Cosmed Quark CPET; Cosmed, Rome, Italy). Further details are given in the ESM Methods.

#### High-resolution respirometry

Mitochondrial respiration was assessed in permeabilised muscle fibres, as previously described [18]. Fibres were permeabilised with saponin (50 µg/ml) in ice-cold BIOPS buffer, then washed in mitochondrial respiration medium (MiR05; see ESM Methods for buffer compositions). Blotted fibres were weighed and transferred to a respirometer (Oxygraph-2k; Oroboros, Innsbruck, Austria) in MiR05 at 37°C. Oxygen was maintained between 300 and 500 µmol/l. Background respiration was assessed before adding substrates and was subtracted from all subsequent values. Leak respiration was assessed with glutamate (10 mmol/l), malate (0.5 mmol/l) and pyruvate (5 mmol/l). NADH-linked respiration was assessed by adding ADP (5 mmol/l) and cytochrome *c* (10 mmol/l). Maximal oxidative phosphorylation (OXPHOS) capacity was assessed after addition of glycerol-3-phosphate (10 mmol/l) and succinate (10 mmol/l). Electron transport system (ETS) capacity was assessed via carbonylcyanide-4-trifluoro-methoxyphenylhydrazone (FCCP) titration (0.5 µmol/l steps) and succinate-linked respiration was measured after addition of rotenone (0.5 µmol/l). Respiration experiments were performed in duplicate or triplicate and averaged. Respiration was normalised to wet weight ([pmol O_2_] s^−1^ mg^−1^). Respirometry was performed on *n*=15 individuals per group due to tissue limitations.

#### Transmission electron microscopy

Samples were prepared for transmission electron microscopy (TEM) as previously described [18]; further details are provided in ESM Methods. Biopsy pieces were fixed in 2.5% wt/vol. glutaraldehyde (24 h), post-fixed in 1% osmium tetroxide and 1.5% wt/vol. potassium ferrocyanide (1 h), washed and dehydrated through graded ethanol incubations. Samples were embedded in Epon812 after impregnation in propylene oxide/Epon812 (1:1, 1 h), then polymerised for 3 days at 65°C. Ultra-thin (60 nm) longitudinal sections were stained with uranyl acetate (3.5% wt/vol., 5 min) and lead citrate (3% wt/vol., 5 min) and visualised using an FEI Tecnai 120kV transmission electron microscope (ThermoFisher Scientific, USA) using a Veleta camera. For each participant, four fibres were imaged, with four subsarcolemmal and four intermyofibrillar images per fibre. Images were taken systematically at the same magnification and analysed with ImageJ (version 1.5; https://imagej.net/ij/download.html). Mitochondrial images were also scored for glycogen and lipid content on a 0–5 scale by three blinded, independent raters as previously described [19]. Capillary images were obtained in a subset of participants (with type diabetes *n*=5; control individuals *n*=7) to assess basement membrane thickness. Due to tissue limitations, TEM data are presented for 16 individuals with type 1 diabetes and 15 control individuals.

#### Capillarisation

Capillarisation was assessed on 10 µm transverse sections stained with *Ulex europaeus* agglutin 1 lectin as previously described [20]. Images were acquired at ×20 magnification (Olympus VS200), and capillary density and capillary/fibre ratio were calculated using ImageJ.

#### Statistical analyses

Data are presented as mean ± SD unless stated otherwise. Normality was assessed via the Shapiro–Wilks test. Group differences were assessed using independent *t* tests or Mann–Whitney *U* tests. Subgroup analyses stratified groups by the median $\dot{V}{\text{O}}_{\text{2max}}$, age, disease duration, HbA_1c_ and BMI, followed by one- or two-way ANOVA with Holm–Šídák post hoc tests. Pearson’s correlations were used to explore the relationships between variables. Analyses were performed in GraphPad Prism v9 (GraphPad Software, USA), with significance set at *p*<0.05.

## Results

Participant characteristics are displayed in Table 1. There were no significant differences for any descriptive variable other than HbA_1c_. The HbA_1c_ values were relatively low, suggesting a well-controlled cohort of individuals with type 1 diabetes. Hence, these findings indicated that the matching of the two groups was successful.

**Table 1:** Cohort characteristics

Characteristic	Control participants	Participants with type 1 diabetes	*p* value
*n*	17	17	
Sex (*n* male/*n* female)	9/8	9/8	
Age (years)	38±16	40±17	0.75
Height (cm)	178±8	178±9	0.87
Weight (kg)	74±14	77±9	0.56
BMI (kg/m^2^)	23±3	24±2	0.26
$\dot{V}{\text{O}}_{\text{2max}}$(l/min)	3.11±0.73	3.06±0.89	0.87
$\dot{V}{\text{O}}_{\text{2max}}$(ml kg^−1^ min^−1^)	41±8	40±10	0.51
Peak power (W)	285±67	287±84	0.93
Peak power (W/kg)	3.85±0.75	3.75±1.00	0.74
Moderate physical activity (min/week)	228±232	231±129	0.96
Intense physical activity (min/week)	173±83	164±133	0.85
HbA_1c_ (mmol/mol)	31.0±2.7	45.9±7.5	<0.001
HbA_1c_ (%)	5.01±0.25	6.37±0.69	<0.001
Disease duration (years)	-	20±16	

Values are displayed as mean ± SD

### Skeletal muscle mitochondrial content and morphology

**Fig. 1:**
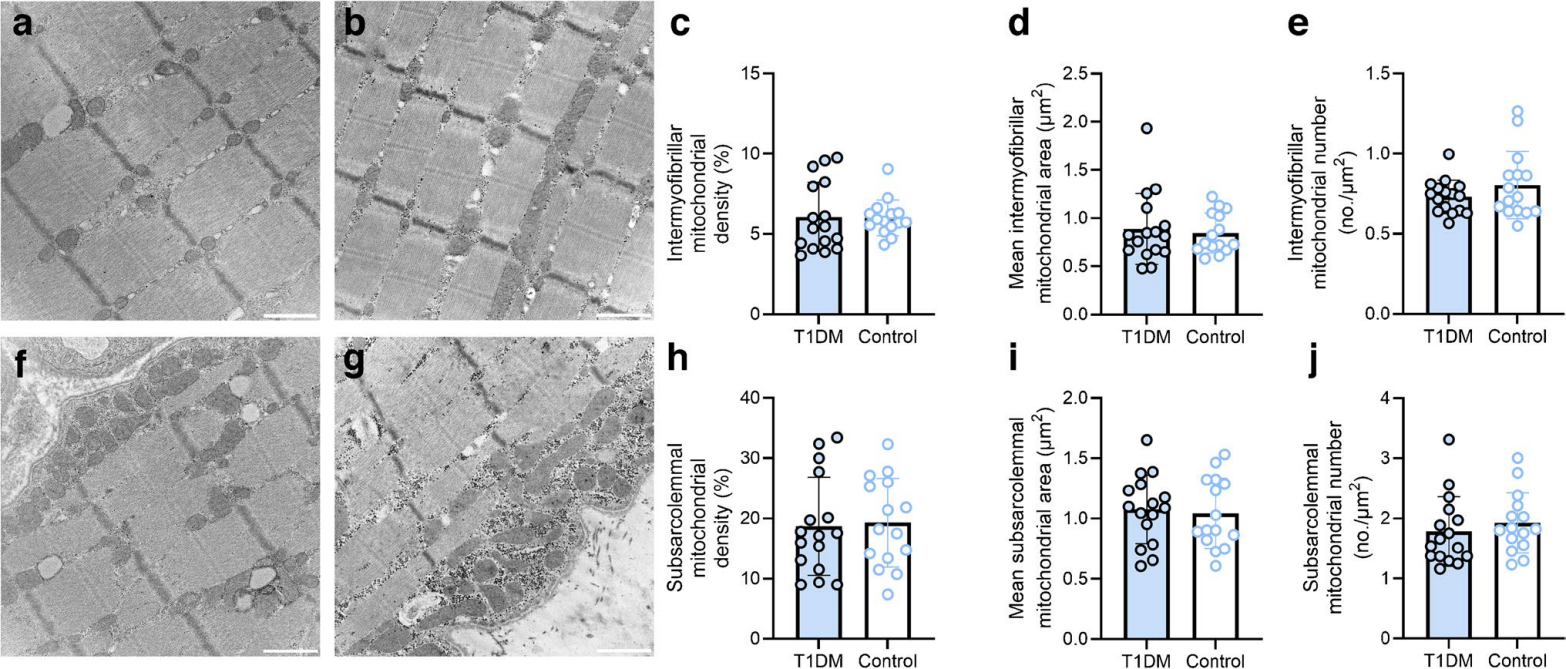
Skeletal muscle mitochondrial density, number and size in individuals with and without type 1 diabetes. (**a**, **b**) Representative images of the intermyofibrillar region in a control participant (**a**) and a participant with type 1 diabetes (**b**). (**c**) Intermyofibrillar mitochondrial density. (**d**) Mean individual intermyofibrillar mitochondrial area. (**e**) Number of intermyofibrillar mitochondria per µm^2^. (**f**, **g**) Representative images of the subsarcolemmal region in a control participant (**f**) and a participant with type 1 diabetes (**g**). (**h**) Subsarcolemmal mitochondrial density. (**i**) Mean individual subsarcolemmal mitochondrial area. (**j**) Number of subsarcolemmal mitochondria per µm^2^. *n*=16 for the type 1 diabetes group, *n*=15 for the control group. Columns and error bars represent means ± SD, circles reflect individual data points. Scale bar, 500 nm. T1DM, type 1 diabetes mellitus

We determined whether individuals with type 1 diabetes displayed altered mitochondrial content and morphology (Fig. 1) by performing TEM on single skeletal muscle fibres in both the subsarcolemmal and intermyofibrillar regions. We manually traced the outlines of 13,345 individual mitochondria within the intermyofibrillar region (Fig. 1a, b and ESM Fig. 2) and 9855 individual mitochondria within the subsarcolemmal region (Fig. 1f, g and ESM Fig. 2) to determine mitochondrial density and morphology. We found that, for both the intermyofibrillar and subsarcolemmal regions, there were no between-group differences in mitochondrial density (Fig. 1c, h), mean individual mitochondrial size (Fig. 1d, i) or number of mitochondria per µm^2^ (Fig. 1e, j). Moreover, there were no between-group differences with respect to any mitochondrial morphological variable (i.e. perimeter, circularity, minimal and maximal Feret’s diameter or roundness; see ESM Fig. 3). These findings indicate that when aerobic training status is accounted for, individuals with type 1 diabetes display similar skeletal muscle mitochondrial density, size and morphology to healthy control individuals.

### Skeletal muscle mitochondrial respiration

**Fig. 2:**
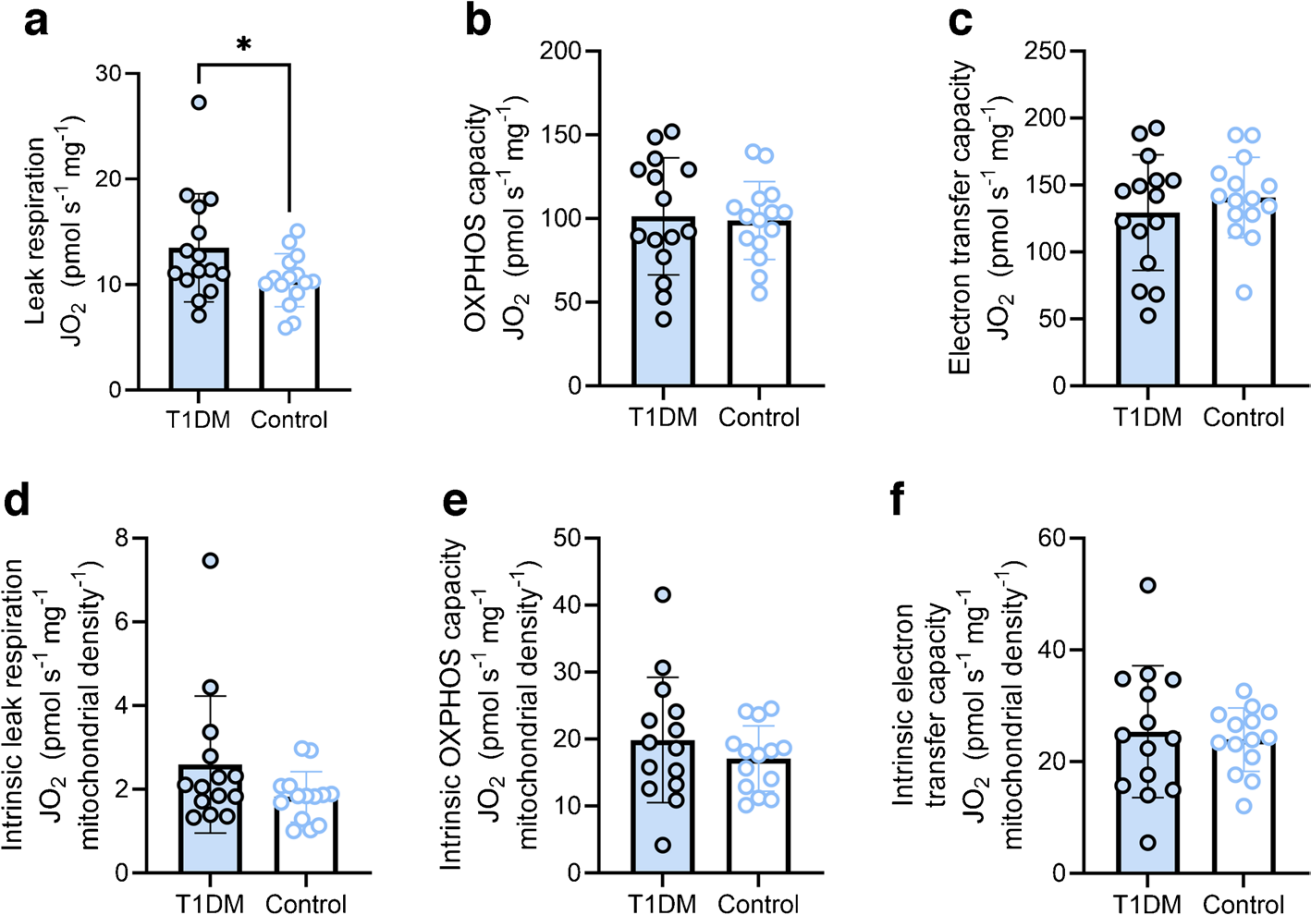
Skeletal muscle mitochondrial respiration in individuals with and without type 1 diabetes. (**a**) Leak respiration in the presence of NADH-linked substrates and no ADP. (**b**) Maximal OXPHOS capacity in the presence of saturating (ADP) and convergent electron flow through complexes I and II. (**c**) Electron transfer capacity measured in the presence of optimal concentrations of uncoupler. (**d**) Intrinsic leak respiration. (**e**) Intrinsic maximal OXPHOS capacity. (**f**) Intrinsic electron transfer capacity. *n*=15 (**a**–**c**) or *n*=14 (**d**–**f**) for each comparison in both groups. Columns and error bars represent means ± SD, circles reflect individual data points. *p<0.05. JO_2_, mitochondrial oxygen consumption; T1DM, type 1 diabetes mellitus

Given the previous work demonstrating impaired skeletal muscle mitochondrial respiration in individuals with type 1 diabetes [8], we assessed mitochondrial respiration in permeabilised fibres (Fig. 2). We found a greater leak respiration in individuals with type 1 diabetes than in those without (Fig. 2a). However, no between-group differences were observed in maximal OXPHOS capacity (Fig. 2b), or maximal electron transfer capacity (Fig. 2c). As differences in absolute values of respiration can be driven by between-sample differences in mitochondrial content, we normalised our respiration values for the unique intermyofibrillar mitochondrial density of each participant to derive intrinsic mitochondrial respiration (i.e. respiration per mitochondrion). When normalising for mitochondrial density, differences in leak respiration were no longer significant (Fig. 2d). Similarly, there were no between-group differences in intrinsic OXPHOS capacity (Fig. 2e) or intrinsic electron transfer capacity (Fig. 2f). Moreover, we also found no differences in mitochondrial succinate dehydrogenase activity between groups, in both high- and low-oxidative fibres (ESM Fig. 4). Collectively, these findings indicate that when $\dot{V}{\text{O}}_{\text{2max}}$ is controlled for, minimal differences in mitochondrial respiration exist between individuals with type 1 diabetes and healthy control individuals.

### Skeletal muscle nutrient accumulation

**Fig. 3:**
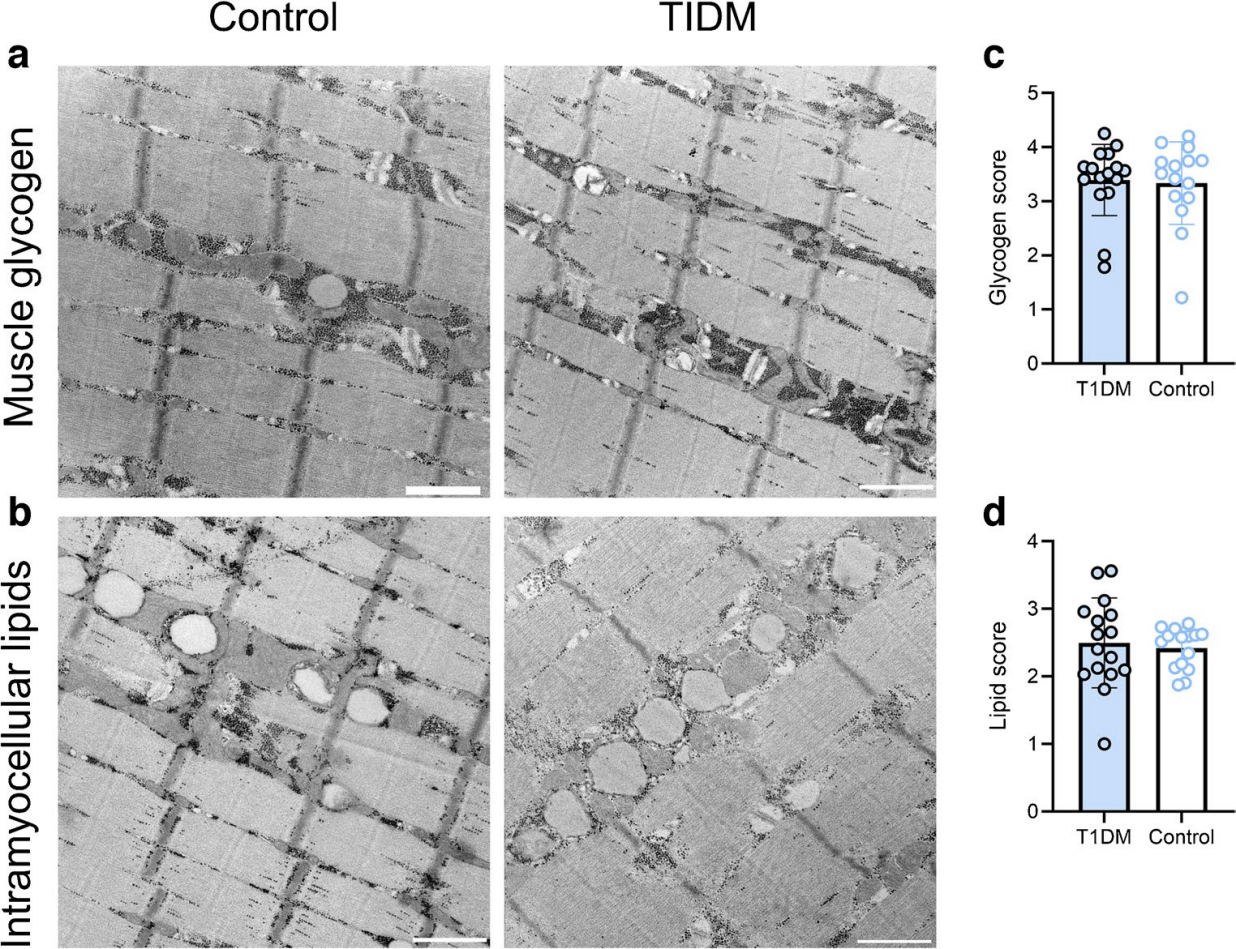
Skeletal muscle nutrient accumulation in individuals with and without type 1 diabetes. (**a**, **b**) Representative images showing skeletal muscle glycogen (**a**) and intramyocellular lipid deposition (**b**) in a control participant and a participant with type 1 diabetes. (**c**, **d**) Muscle glycogen score (**c**) and intramyocellular lipid deposition score (**d**) in the type 1 diabetes and control groups. *n*=16 (type 1 diabetes group) or *n*=15 (control group). Nine hundred and twenty-six images were scored in a systematic, blinded fashion by three experienced raters, with the score for each participant reflecting the mean across all images from that participant. Lipid score was correlated with mitochondrial fragmentation in control participants (*r*=0.68, *p*=0.0057) but not in participants with type 1 diabetes (*r*=−0.34, *p*=0.20). Columns and error bars represent means ± SD, circles reflect individual data points. Scale bar, 500 nm. T1DM, type 1 diabetes mellitus

We previously found that fragmentation of the mitochondrial network occurs alongside intramuscular lipid accumulation following bed rest [19] and in human ageing [18], and intracellular lipid accumulation can have lipotoxic effects on mitochondria [21]. A link between intramuscular lipid accumulation in type 1 diabetes, insulin resistance and mitochondrial dysfunction was recently proposed [22]; however, human data addressing this link are lacking. Hence, we tested whether individuals with type 1 diabetes displayed altered muscle glycogen and lipid accumulation. Three independent, blinded raters scored the images used for mitochondrial analysis (i.e. a total of 926 images) on a scale from 0 (no glycogen, no lipid) to 5 (extreme lipid and glycogen accumulation). Typical examples of muscle glycogen and intramyocellular lipids from each group are shown in Fig. 3a, b. Both muscle glycogen accumulation (Fig. 3c) and intramyocellular lipid content (Fig. 3d) did not differ between the two groups. Hence, these findings imply that intramuscular lipid or glycogen accumulation did not differ between individuals with well-managed type 1 diabetes compared with healthy control individuals.

### Skeletal muscle microvasculature

**Fig. 4:**
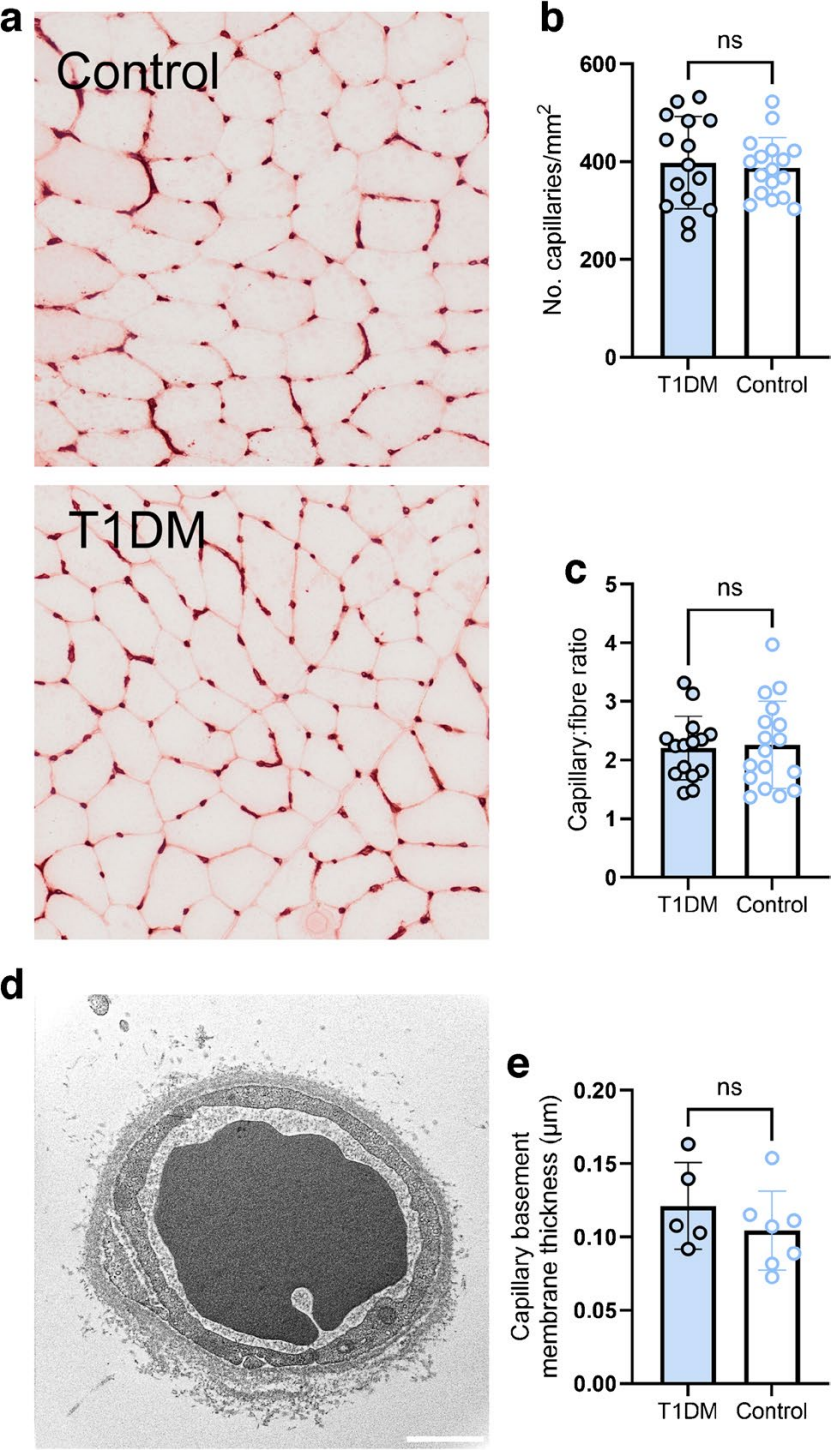
Skeletal muscle capillarisation in control individuals (*n*=17) and individuals with type 1 diabetes (*n*=15). (**a**) Representative images used for quantification of skeletal muscle capillarisation in a healthy control participant and a participant with type 1 diabetes. Images were acquired at ×20 magnification. (**b**, **c**) Capillary density (**b**) and capillary-to-fibre ratio (**c**) in the type 1 diabetes and control groups. (**d**) Representative electron micrograph used for quantification of skeletal muscle capillary basement membrane thickness. (**e**) Skeletal muscle capillary basement membrane thickness in control participants (*n*=7) and participants with type 1 diabetes (*n*=5). Columns and error bars represent means ± SD, circles reflect individual data points. Scale bar, 500 nm. ns, no significant difference; T1DM, type 1 diabetes mellitus

Given that long-term type 1 diabetes may result in capillary damage and rarefaction, and that alterations in muscle capillarisation can precede mitochondrial alterations in response to bed rest [23], we wondered whether the microvascular alterations commonly associated with this condition may have already occurred in this cohort of individuals with well-managed type 1 diabetes. To this end, we determined markers for capillarisation in skeletal muscle sections (Fig. 4a). We found no differences between groups regarding skeletal muscle capillary density (Fig. 4b) or the capillary/fibre ratio (Fig. 4c). Neither variable correlated with disease duration (both *p*>0.39) or HbA_1c_ values (both *p*>0.16). It has also previously been demonstrated that individuals with type 1 diabetes display a thickening of the muscle capillary basement membrane [24], which could increase resistance to blood–mitochondria O_2_ flux and thereby hamper mitochondrial respiration. In a subset of individuals (*n*=5 type 1 diabetes, *n*=7 controls), we obtained high-magnification images of capillaries using TEM (Fig. 4d). We found no differences in capillary basement membrane thickness between individuals with type 1 diabetes and healthy control individuals (Fig. 4e). We therefore found no evidence of skeletal muscle microvascular alterations in this cohort of individuals with type 1 diabetes, similar to other recent studies [25].

### Predictors of mitochondrial health in type 1 diabetes

**Fig. 5:**
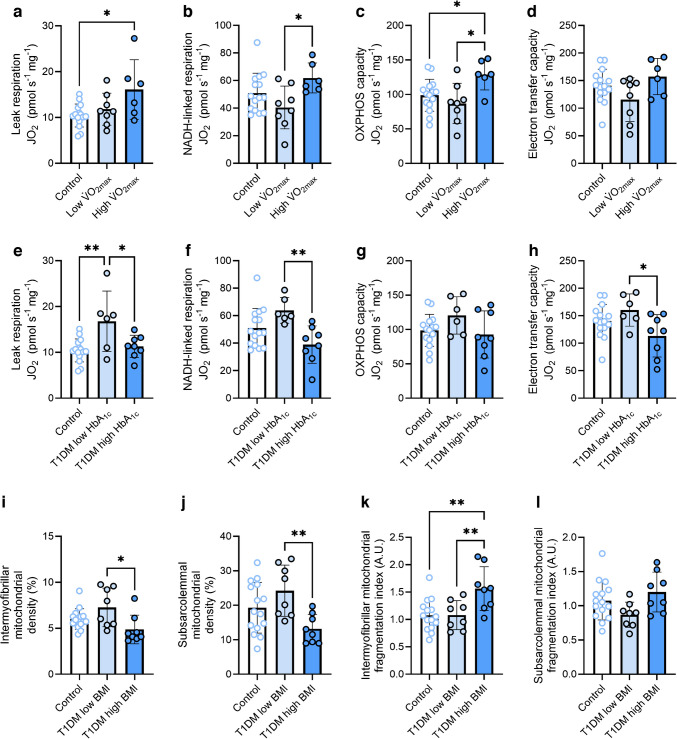
Mitochondrial characteristics in healthy control individuals and individuals with type 1 diabetes, with the type 1 diabetes group split by maximal oxygen uptake (**a**–**d**), HbA_1c_ concentrations (**e**–**h**) and BMI (**i**–**l**). (**a**, **e**) Leak respiration in the presence of NADH-linked substrates and no ADP. (**b**, **f**) NADH-linked respiration in the presence of NADH-linked substrates and saturating ADP. (**c**, **g**) Maximal OXPHOS capacity in the presence of saturating ADP and convergent electron flow through complexes I and II. (**d**, **h**) Electron transfer capacity measured in the presence of optimal concentrations of uncoupler. (**i**) Intermyofibrillar mitochondrial density. (**j**) Subsarcolemmal mitochondrial density. (**k**) Intermyofibrillar mitochondrial fragmentation index. (**l**) Subsarcolemmal mitochondrial fragmentation index. (**a**–**d**) *n*=15 control participants, *n*=8 participants with type 1 diabetes and low $\dot{V}{\text{O}}_{\text{2max}}$, *n*=6 participants with type 1 diabetes and high $\dot{V}{\text{O}}_{\text{2max}}$. (**e**–**h**)* n*=15 control participants, *n*=6 participants with type 1 diabetes and low HbA_1c_, *n*=8 participants with type 1 diabetes and high HbA_1c_. (**i**–**l**) *n*=15 control participants, *n*=8 participants with type 1 diabetes and low BMI, *n*=8 participants with type 1 diabetes and high BMI. *p<0.05, **p<0.001. A.U., arbitrary units; JO_2_, mitochondrial oxygen consumption; T1DM, type 1 diabetes mellitus

**Fig. 6:**
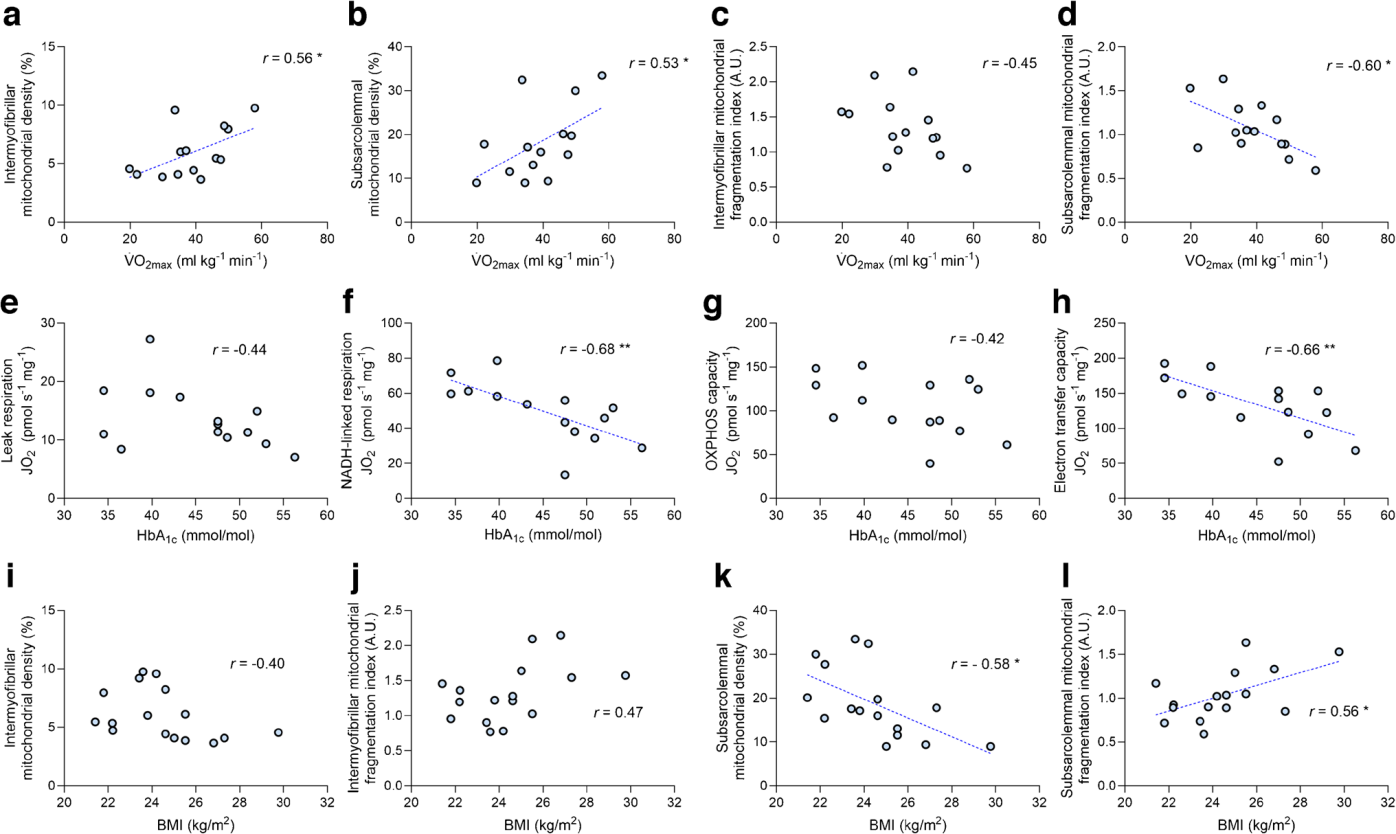
Correlates of mitochondrial respiration, density and fragmentation in individuals with type 1 diabetes. (**a**–**d**) Correlations between maximal oxygen uptake ($\dot{V}{\text{O}}_{\text{2max}}$) and intermyofibrillar mitochondrial density (**a**), subsarcolemmal mitochondrial density (**b**), intermyofibrillar mitochondrial fragmentation index (**c**) and subsarcolemmal mitochondrial fragmentation index (**d**). (**e**–**h**) Correlations between HbA_1c_ and leak respiration (**e**), NADH-linked respiration (**f**), OXPHOS capacity (**g**) and electron transfer capacity (**h**). (**i**–**l**) Correlations between BMI and intermyofibrillar mitochondrial density (**i**), intermyofibrillar mitochondrial fragmentation index (**j**), subsarcolemmal mitochondrial density (**k**) and subsarcolemmal mitochondrial fragmentation index (**l**). *n*=16 (**a**–**d**, **i**–**l**) or *n*=14 participants (**e**–**h**). Circles reflect individual participant data whereas dashed lines indicate line of best fit for significant correlations. *p<0.05, **p<0.001. A.U., arbitrary units; JO_2_, mitochondrial oxygen consumption; T1DM, type 1 diabetes mellitus

To determine which factors were important for shaping mitochondrial content and respiration in type 1 diabetes, we performed a subgroup analysis of this cohort. The type 1 diabetes group was split according to age, disease duration, $\dot{V}{\text{O}}_{\text{2max}}$, BMI, HbA_1c_ values and sex using the median value to stratify two groups for each variable. The details relating to each group derived for this analysis are given in ESM Tables 1 and 2. No variables relating to mitochondrial respiration, density, number, size, or fragmentation were impacted by age (ESM Fig. 5) or disease duration (ESM Fig. 6). Although, generally, mitochondrial density and respiration were lower and fragmentation was higher in female participants than in male participants, this effect did not depend on the presence or absence of type 1 diabetes (no interaction effects, ESM Fig. 7). However, individuals with type 1 diabetes and a lower $\dot{V}{\text{O}}_{\text{2max}}$ displayed lower NADH-linked respiration (Fig. 5b) and OXPHOS capacity (Fig. 5c) than those with type 1 diabetes and a higher $\dot{V}{\text{O}}_{\text{2max}}$. Similarly, participants with type 1 diabetes and a lower HbA_1c_ displayed greater leak respiration (Fig. 5e), NADH-linked respiration (Fig. 5f) and electron transfer capacity (Fig. 5h) than those with type 1 diabetes and a higher HbA_1c_. Mitochondrial density and fragmentation of both muscle regions did not differ between individuals with lower and higher $\dot{V}{\text{O}}_{\text{2max}}$ (ESM Fig. 8a–d) or lower and higher HbA_1c_ (ESM Fig. 8e–h), implying that greater aerobic fitness and glycaemic control are associated with improved mitochondrial respiration in type 1 diabetes without alterations in mitochondrial quantity or fragmentation. Conversely, individuals with type 1 diabetes and a low BMI displayed no differences in mitochondrial respiratory function when compared with those with a high BMI (ESM Fig. 8i–l) but displayed greater mitochondrial densities (Fig. 5i, j) and less intermyofibrillar mitochondrial fragmentation (Fig. 5k). Finally, we explored correlations between $\dot{V}{\text{O}}_{\text{2max}}$, HbA_1c_, BMI and mitochondrial variables in the type 1 diabetes group (Fig. 6). We found that $\dot{V}{\text{O}}_{\text{2max}}$ related positively with subsarcolemmal mitochondrial density (Fig. 6b) and was inversely related to the subsarcolemmal mitochondrial fragmentation index (Fig. 6d). HbA_1c_ was inversely associated with NADH-linked respiration (Fig. 6f) and electron transfer capacity (Fig. 6h), whereas BMI was inversely related to subsarcolemmal mitochondrial density (Fig. 6k) and positively related to subsarcolemmal mitochondrial fragmentation (Fig. 6l). Collectively, these findings suggest that modifiable lifestyle factors such as $\dot{V}{\text{O}}_{\text{2max}}$ (see also ESM Fig. 9), BMI and glucose management play an important role in shaping mitochondrial health in individuals with type 1 diabetes.

## Discussion

This study demonstrates that when aerobic fitness is controlled for, skeletal muscle mitochondrial alterations in type 1 diabetes are more subtle than previously suggested. Participants with type 1 diabetes and control participants with similar $\dot{V}{\text{O}}_{\text{2max}}$ displayed no differences in mitochondrial density, number, size or fragmentation in either intermyofibrillar or subsarcolemmal regions. Leak respiration was slightly increased in type 1 diabetes but normalised when adjusted for mitochondrial density. Both maximal oxidative phosphorylation capacity and electron transfer capacity were comparable between groups. We also observed no alterations in capillarisation, basement membrane thickness or intramuscular glycogen and lipid content. Subgroup analyses indicated that better glucose management was linked to higher mitochondrial respiration, whereas higher BMI was linked with lower mitochondrial density and greater fragmentation. Collectively, these findings underscore the role of maintaining a high aerobic capacity, good glucose management and healthy BMI in protecting skeletal muscle mitochondrial health in type 1 diabetes.

### Skeletal muscle mitochondrial respiratory function in type 1 diabetes

Previous studies using ^31^P-MRS have reported impaired in vivo mitochondrial bioenergetic function in young, otherwise healthy, individuals with type 1 diabetes [4–6]. More recent studies have shown reductions in ex vivo mitochondrial respiration in permeabilised skeletal muscle fibres [8], sex-specific mitochondrial bioenergetic decrements in male participants [7] and a 30% reduction in skeletal muscle mitochondrial density in older participants with type 1 diabetes [9]. However, many previous studies used sedentary participants [4, 6] and most did not include a gold-standard indicator of aerobic fitness [5, 7–9, c.f. 26]. A key strength of the present study in relation to this previous work is therefore the matching of our participants with type 1 diabetes to control participants according to $\dot{V}{\text{O}}_{\text{2max}}$. $\dot{V}{\text{O}}_{\text{2max}}$ is strongly related to physical activity levels [27], is considered a gold-standard in the assessment of cardiorespiratory fitness [28] and is an independent predictor of all-cause mortality and CVD risk [29]. Additionally, the cohort described herein possessed better glucose management than those previously reporting mitochondrial impairments in this population (i.e. HbA_1c_, present study: 45.9 mmol/mol [6.4%]; Monaco et al [8]: 63.0 mmol/mol [7.9%]; Heyman et al [11] 67 mmol/mol [8.3%]). Our findings partially conflict with those of Minnock et al [26], who observed lower mitochondrial complex I, III and V protein content in individuals with type 1 diabetes compared with healthy control individuals with a similar $\dot{V}{\text{O}}_{\text{2max}}$. However, in that study the mean $\dot{V}{\text{O}}_{\text{2max}}$ of the diabetes group was lower (32 vs 40 ml kg^−1^ min^−1^) and their glucose management was less optimal (i.e. HbA_1c_, 63 mmol/mol [7.9%] vs 45.9 mmol/mol [6.4%]) than that reported herein, which likely explains the discrepant findings. Hence, our data suggest that when comparisons are performed at a similar $\dot{V}{\text{O}}_{\text{2max}}$, and in participants with well-controlled diabetes, alterations in muscle mitochondrial respiration, content and morphology in individuals with type 1 diabetes may be less pronounced than previously thought.

Despite the similarities between groups in terms of mitochondrial respiration, density, number and size, leak respiration was elevated in the type 1 diabetes group. The enhanced leak respiration is consistent with previous observations of a 40% elevation in leak respiration in older individuals with type 1 diabetes [9]. However, in the present study, the between-group difference in leak respiration was abolished when respiratory values were normalised by mitochondrial density, suggesting that the intrinsic proton leak across the inner mitochondrial membrane was not different but that small, non-significant differences in mitochondrial content might explain the difference in leak respiration normalised to tissue wet weight. Moreover, individuals with type 1 diabetes and a low HbA_1c_ displayed a greater leak respiration than those with higher HbA_1c_, despite greater NADH-linked respiration and electron transfer capacity. Hence, the finding of an elevated leak respiration may not be reflective of a negative bioenergetic adaptation. Indeed, the higher leak respiration appeared to be inconsequential for whole-body exercise capacity, which was the same between groups. Collectively, these findings suggest no intrinsic mitochondrial respiratory impairment in type 1 diabetes, independent from cardiorespiratory fitness and/or physical activity status.

### Skeletal muscle nutrient accumulation

Excessive accumulation of lipid, particularly within the vicinity of mitochondria, can cause lipid peroxidation and damage to mitochondrial proteins [21]. Moreover, accumulation of intramuscular lipid precedes the loss of mitochondrial content and oxidative capacity that occurs in insulin-resistant muscle [21]. In support of this, overnight lipid infusion in healthy humans induces muscle mitochondrial fragmentation [30] and muscle lipid accumulation precedes the reduction in mitochondrial respiratory function that develops during prolonged bed rest [19]. Indeed, it has recently been proposed that intramuscular lipid deposition lies at the nexus between altered mitochondrial metabolism and insulin resistance in type 1 diabetes [22], with evidence supporting aberrant intramyocellular lipid accumulation in animal models of type 1 diabetes [31]. In the present study, however, we found no evidence of altered muscle glycogen or lipid deposition in individuals with well-managed type 1 diabetes. Human evidence pertaining to intramyocellular lipid accumulation in type 1 diabetes is sparse, with some studies indicating elevated intramyocellular lipid content in individuals with type 1 diabetes compared with healthy individuals [32, 33], whereas others found no differences [4, 6]. Our data demonstrate that in individuals with well-managed type 1 diabetes and normal exercise capacity, no differences in either skeletal muscle lipid or glycogen deposition were apparent. Hence, aggravated intramyocellular lipid accumulation is likely not an obligatory complication of type 1 diabetes.

### Limitations

A limitation of this study was the lack of objective physical activity measures (e.g. accelerometery). Individuals with type 1 diabetes may require more exercise to attain a given exercise capacity [26], and previous studies have reported mitochondrial alterations in type 1 diabetes despite matched activity levels [4, 8]. Future studies involving well-controlled exercise training interventions and objective activity measurements are required to verify this. Another limitation is that we did not assess mitochondrial respiration at submaximal ADP concentrations or perform complex IV-specific assays, conditions under which respiration has been shown to be impaired in previous studies [8, 11]. However, the absence of group differences across most mitochondrial outcomes suggests preserved mitochondrial health in our well-controlled type 1 diabetes cohort. Finally, the subgroup analyses were exploratory and not part of the original study design. These findings should therefore be interpreted cautiously, although they align with prior research implicating $\dot{V}{\text{O}}_{\text{2max}}$, BMI and HbA_1c_ as determinants of mitochondrial health in healthy individuals and individuals with diabetes. Future studies with larger cohorts will be needed to confirm these trends.

### Perspectives and conclusions

Previous reports [4, 6–10] of impaired skeletal muscle mitochondrial health in individuals with type 1 diabetes have raised clinical concerns, given the role of skeletal muscle mitochondria in glucose metabolism and overall metabolic health. The findings of the present study challenge the notion that type 1 diabetes per se is associated with an intrinsic deficit in mitochondrial health. Specifically, when comparisons were performed between individuals with type 1 diabetes and healthy control individuals possessing the same $\dot{V}{\text{O}}_{\text{2max}}$, the majority of differences were abolished. Whilst we were unable to investigate all mitochondrial functions in the present study, our data indicate that under the specific conditions tested, there was no evidence of any mitochondrial impairment in physically active individuals with well-managed type 1 diabetes.

Additional lifestyle factors, such as glucose control and BMI, exerted additional influence on the mitochondrial variables measured in the present study, whereas age, sex and disease duration did not influence any mitochondrial outcome. These findings highlight the role of modifiable lifestyle factors for maintaining mitochondrial health in type 1 diabetes, as opposed to immutable characteristics such as age and disease duration. We show that maintaining a normal exercise capacity, good glucose management and a healthy BMI can enable individuals with type 1 diabetes to maintain their skeletal muscle mitochondrial health to a degree similar to that seen in healthy individuals without diabetes. Hence, optimal insulin therapy, proper nutritional management and regular exercise appear to be suitable strategies to help individuals with type 1 diabetes to maintain or improve their mitochondrial health. It has been proposed that type 1 diabetes represents an accelerated skeletal muscle ageing phenotype [34]; however, the present findings imply that such a phenotype may be largely abrogated by maintaining a healthy BMI, good glucose management and undertaking regular exercise.

In conclusion, our findings indicate that with well-controlled diabetes and lifestyle care, skeletal muscle mitochondrial health in people with type 1 diabetes can be maintained. Specifically, this study demonstrates that the majority of mitochondrial differences between individuals with well-controlled type 1 diabetes were abolished when healthy control individuals were matched for $\dot{V}{\text{O}}_{\text{2max}}$, age, sex, BMI and physical activity. The previously reported mitochondrial impairments in type 1 diabetes can thus likely be explained by a lower exercise capacity, less-well-managed diabetes (i.e. higher HbA_1c_) and potentially concomitant adiposity. Ultimately, this study underscores the importance of lifestyle factors in maintaining mitochondrial health in people with type 1 diabetes, and in particular, nutrition, exercise and glucose control.

## Supplementary Information

Below is the link to the electronic supplementary material.

ESM (PDF 1.68 MB)

## Data Availability

Data are available upon request from the corresponding authors.

## Abbreviations

OXPHOS: Oxidative phosphorylation
TEM: Transmission electron microscopy
$\dot{V}{\text{O}}_{\text{2max}}$: Maximal oxygen uptake
