# Supplementary material for: Skeletal muscle mitochondrial health in type 1 diabetes: the role of exercise capacity and lifestyle factors
Source: Diabetologia. 2025 May 21;68(8):1823–35. doi: 10.1007/s00125-025-06451-1 (PMC12246007; doi:10.1007/s00125-025-06451-1)
Supplement: Supplementary file 1 — ESM (PDF 1.68 MB) [file 125_2025_6451_MOESM1_ESM.pdf]

ELECTRONIC SUPPLEMENTARY MATERIAL FOR SKELETAL MUSCLE MITOCHONDRIAL HEALTH IN TYPE 1 DIABETES: THE ROLE OF EXERCISE CAPACITY AND LIFESTYLE FACTORS

Richie P. Goulding[1], Braeden T. Charlton[1], Ellen A. Breedveld[1], Jelle Huijts[1], Matthijs van der Laan[1], Anne R. Strating[1], Wendy Noort[1], Aryna Kolodyazhna[1], Anita E. Grootemaat[2], Frank W. Bloemers[3], Nicole N. van der Wel[2], Rob C.I. Wüst[1]

[1]Department of Human Movement Sciences, Faculty of Behavioural and Movement Sciences, Vrije Universiteit Amsterdam, Amsterdam Movement Sciences, Amsterdam, Netherlands
[2]Electron Microscopy Centre Amsterdam, Amsterdam UMC, Location Academic Medical Centre, Amsterdam, Netherlands
[3]Department of Trauma Surgery, Amsterdam Movement Sciences, Amsterdam UMC, Vrije Universiteit Amsterdam, Amsterdam, Netherlands.

Running title: Skeletal muscle mitochondria in type 1 diabetes

Corresponding authors: Richie Goulding and Rob CI Wüst, Department of Human Movement Sciences, Vrije Universiteit Amsterdam, Van der Boechorststraat 7, 1081 BT Amsterdam, Netherlands. E-mail address: r.p.goulding@vu.nl, r.wust@vu.nl.

EXTENDED METHODS

Participants

Seventeen individuals with type 1 diabetes and seventeen healthy controls matched for age, sex, body mass index, physical activity levels, and $\dot{V}O_{2max}$ participated. The experiment was approved by the Medical Ethics Committee of Vrije Universiteit Medical Center (VUmc), and conformed to the Declaration of Helsinki. The study was registered with the Netherlands National Trial Register (trial number: NL9583). Details on participant screening and recruitment are given in ESM Figure 1. Our power calculations were performed using mitochondrial respiration as a primary outcome, an expected group difference of 30% [1], a within-group standard deviation of 24%, a power of 80%, and a two-tailed alpha of 0.05. This yielded a sample size of approximately 11 per group. We aimed to recruit seventeen per group to account for dropouts and to achieve sufficient statistical power for additional analyses.

Experimental Overview

Each participant visited the laboratory on two separate occasions. At the initial visit, participants completed an incremental ramp exercise test on a cycle ergometer (Lode Excalibur Sport, Lode, Groningen, The Netherlands) for determination of $\dot{V}O_{2max}$, exercise capacity, and other variables related to *in vivo* aerobic function. For the second visit, a vastus lateralis muscle biopsy was taken. Muscle biopsies were taken from the vastus lateralis at one-third of the distal length using a suction-supported 5 mm Bergström needle (Pelomi, Albertslund, Denmark). The biopsy site was locally anaesthetized using 2% lidocaine solution, and a ~1 cm incision was made through the skin. Biopsy samples were carefully removed and partitioned into three pieces. One ~30 mg piece was aligned according to the fibre arrangement under a light microscope and frozen in liquid nitrogen for histochemistry, another ~5 mg piece was placed in fixative solution for later TEM analysis, and another ~15 mg piece was utilized for high-resolution respirometry experiments. For histochemistry, frozen muscle samples were cut into 10 µm thick sections in transverse orientation in a cryostat at -20°C, before being mounted on polylysine-coated slides. Prior to the first visit, participants completed a 7 day physical activity questionnaire. Prior to all visits, participants were instructed to arrive 3 hours postprandial, having avoided alcohol and strenuous exercise for the preceding 24 hours, and having avoided caffeine on the day of the visit. Prior to the biopsy, participants with type 1 diabetes were instructed to omit their fast-acting insulin but maintain their basal insulin; this was primarily

for a companion study on muscle glucose signalling, the results of which are not reported here. Similarly, participants with type 1 diabetes were instructed to arrive at the laboratory with blood glucose concentrations between 7-11 $mmol.L^{-1}$ in accordance with exercise safety guidelines for people with type 1 diabetes [2]. Mean blood [glucose] immediately prior to the biopsy was 8.8 ± 2.2 $mmol.L^{-1}$ for the type 1 diabetes group and 5.3 ± 0.4 $mmol.L^{-1}$ for the controls.

Exercise testing procedures

Body mass and height were first measured, followed by measurement of glycated haemoglobin (HbA_{1c}) via a capillary blood sample obtained from the fingertip using a portable analyser ($HbA_{1c}Now^{+}$, PTS Diagnostics, USA). All exercise tests were performed in the same temperature controlled laboratory (18-21 °C and 50 ± 10 % humidity). After the assessment of anthropometric characteristics and instrumentation, the measurements began with a 2 minute period of quiet rest with the participant seated on the ergometer. The test began with a 4 minute period of baseline cycling at 30-40 W, followed by a ramped, linear increase in power output at a rate of 20-30 $W.min^{-1}$ until the limit of tolerance was reached. The individual ramp rates were selected based upon the subject's anthropometric characteristics and self-reported levels of physical activity, with the aim to reach an exercise duration of 8-12 minutes prior to task failure. Participants were instructed to maintain their cadence between 70-90 revolutions/minute, and task failure was defined as the point at which cadence dropped <60 revolutions/minute despite strong verbal encouragement. Pulmonary gas exchange and ventilation were measured on a breath-by-breath basis throughout the entire test using open-circuit spirometry (Cosmed Quark CPET; Cosmed, Rome, Italy). Before each test, the gas analyser and volume transducer were calibrated using gases of known concentrations and a 3-litre syringe and according to the manufacturer's instructions, respectively. Peak power output was determined as the highest power output attained during the test. $\dot{V}O_{2max}$ was determined as the highest 30 second rolling average value recorded during the test. The gas exchange threshold (GET) was determined as an increase in $\dot{V}CO_2$ with respect to $\dot{V}O_2$, an increase in the ventilatory equivalent for $\dot{V}O_2$ without a corresponding increase in the ventilatory equivalent for $\dot{V}CO_2$, and an increase in the end-tidal O_2 pressure without a corresponding increase in end-tidal CO_2 pressure, with correction for the mean response time of $\dot{V}O_2$ kinetics, as previously described [3]. In total, 58% of participants attained the primary criterion of a plateau in the $\dot{V}O_2$ response despite increasing external power output, and end-exercise blood [lactate] (type 1

diabetes: 12.8 ± 3.5 mmol.L^{-1}, controls: 14.5 ± 2.4 mmol.L^{-1}, P = 0.15), respiratory exchange ratio (RER, type 1 diabetes: 1.24 ± 0.10, controls: 1.23 ± 0.06, P = 0.28) and heart rate (type 1 diabetes: 175 ± 21 beats.min^{-1}, controls: 182 ± 11 beats.min^{-1}, P = 0.24) all met the arbitrary thresholds comprising the secondary criteria. Regarding the subjects which displayed a plateau versus those that did not, no differences were found with respect to $\dot{V}O_{2max}$ (plateau: 42.8 ± 9.0 mL.kg^{-1}.min^{-1}, no plateau: 38.4 ± 9.0 mL.kg^{-1}.min^{-1}, P = 0.17), nor the percentage of $\dot{V}O_{2max}$ at which the GET (plateau: 70.2 ± 6.9%, no plateau: 73.6 ± 6.1%, P = 0.15) or RCP (plateau: 87.8 ± 8.3%, no plateau: 89.7 ± 5.2%, P = 0.48) occurred. We contend that if the participants not evincing a plateau in $\dot{V}O_2$ towards the end of the incremental test did so because of submaximal effort, the submaximal thresholds that occur during the test (i.e., GET and RCP) would have occurred at higher percentages of $\dot{V}O_{2max}$; however, this was not what we saw in our dataset. Similarly, the secondary criteria such as end-exercise blood [lactate] (plateau: 13.3 ± 2.8 mmol.L^{-1}, no plateau: 13.8 ± 4.0 mmol.L^{-1}, P = 0.69), RER (plateau: 1.24 ± 0.08, no plateau: 1.22 ± 0.06, P = 0.28) and heart rate (plateau: 181 ± 16 beats.min^{-1}, no plateau: 175 ± 20 beats.min^{-1}, P = 0.69) did not differ between those displaying a plateau and those that did not. Hence, we are confident that for all participants in the present study, $\dot{V}O_{2max}$ was accurately measured.

High-resolution respirometry

Mitochondrial respiration was assessed in permeabilized skeletal muscle fibres as described previously [7]. Muscle fibres were dissected free of connective tissue, fat tissue and blood using sharp forceps. Small bundles of freshly isolated fibres were then placed in ice-cold relaxing buffer (BIOPS) containing 2.7 mM CaK_2EGTA, 7.23 mM K_2EGTA, 5.77 mM Na_2ATP, 6.56 mM $MgCl_2 \cdot 6H_2O$, 20mM taurine, 15mM Na_2Phosphocreatine, 20mM imidazole, 0.5 mM dithiothreitol, 50 mM MES, with a pH of 7.1. Saponin (50 µg.mL^{-1}) was then added to the BIOPS buffer to permeabilize the outer-cell membranes. After this, the fibres were transferred to respiration medium (MiR05) containing: 0.5 mM EGTA, 3.0 mM $MgCl_2 \cdot 6H_2O$, 60 mM K-lactobionate, 20 mM taurine, 10 mM KH_2PO_4, 20 mM HEPES, 110 mM sucrose, 1 g.L^{-1} BSA, with pH of 7.1. Muscle fibres were blotted dry, weighed and transferred to a respirometer (Oxygraph-2k, Oroboros, Innsbruck, Austria) in respiration medium at 37°C. Oxygen concentration was between 300-500 µM throughout all experiments to avoid limitations in oxygen supply (i.e., an approximate PO_2 of 175-300 mmHg). Background respiration was assessed before adding substrates and was subtracted from all subsequent values. Leak respiration was assessed after the addition of sodium glutamate (10 mM), sodium malate (0.5

mM), and sodium pyruvate (5 mM). NADH-linked respiration was assessed after the addition of 5 mM ADP and 10 mM cytochrome c to account for outer mitochondrial membrane integrity. No sample displayed an increase of greater than 10% following cytochrome c addition. Maximal oxidative phosphorylation (OXPHOS) capacity, with convergent electron input via complexes I and II, was assessed after the addition of 10 mM succinate and 10 mM glycerol-3-phosphate. Electron transport system (ETS) capacity was determined via titration of carbonylcyanide-4-trifluoro- methoxyphenylhydrazone (FCCP) in 0.5 µM steps until no further increase in oxygen consumption was observed. Succinate-linked respiration (S+ROT) was measured following inhibition of mitochondrial complex I via the addition of 0.5 µM rotenone. Respiration experiments were performed in duplicate or triplicate depending on tissue availability, and the results were averaged. Respiration values were normalized to wet weight and expressed in pmol $O_2.s^{-1}.mg^{-1}$.

Two separate approaches were employed to assess intrinsic mitochondrial respiration (i.e. mitochondrial quality), independent of mitochondrial abundance. Firstly, values for respiration in each respiratory state outlined above (i.e. leak, NADH-linked, OXPHOS, ETS, succinate-linked respiration) were normalized to the unique intermyofibrillar mitochondrial area density calculated from the TEM analysis for each individual. Because of tissue scarcity, this analysis was only performed in n = 14 individuals per group. The second approach was to calculate various respiratory control ratios/factors from respiration values in the various respiratory states that were measured. As the measurements in each respiratory state are made in the same sample, and thus the same population of mitochondria, they can be utilized to provide insight into aspects of intrinsic mitochondrial respiratory function.

Transmission electron microscopy

Samples were fixed in 2.5% glutaraldehyde solution containing 0.1 M sodium cacodylate buffer (pH 7.3) for 24 h at room temperature and then transferred to storage buffer until later analysis. Samples were subsequently rinsed with ddH_2O for 10 minutes, before post-fixation with 1% osmium tetroxide and 1.5% potassium ferrocyanide in ddH_2O for 60 minutes at room temperature. Following post-fixation, samples were rinsed for 10 minutes with ddH_2O, and dehydrated via a series of 15 minute incubations with a graded series of alcohol concentrations (i.e. 2x70, 80, 90% and 2x100% ethanol). Following dehydration, samples were impregnated with a 1:1 propylene oxide/Epon812 mixture for 1 h at room temperature, before being impregnated with Epon812 at 37°C for 30 min. Finally, samples were embedded longitudinally in moulds containing fresh Epon812 and left to polymerize for 3 days at 65°C. Ultra-thin (10

nm) longitudinal sections cut using a Leica Ultracut EM UC7 and collected on 150-mesh Formavar-coated copper grids and contrasted by staining for 5 min with 3.5% (w/v) uranyl acetate and 5 min with 3% (w/v) lead citrate. Visualisation was performed with a FEI Tecnai 120kV Transmission electron microscope (TEM, ThermoFisher Scientific, Waltman, Massachusetts, VS) using a Veleta camera Plus. Due to limited tissue availability, TEM data were only available for n = 16 individuals with type 1 diabetes and n = 15 healthy control participants. Four individual fibres were imaged per subject, with four images of the subsarcolemmal region and four images of the intermyofibrillar region being taken per fibre, resulting in approximately 16 images per subject per region, or 32 images across both regions per subject, for the analysis of individual mitochondrial area (in μm^2), mitochondrial number (as number.μm^{-2}), and mitochondrial area density (percentage muscle area occupied by mitochondria). Images were taken in a randomized, systematic fashion, at a magnification of 18,500x for quantification of mitochondrial area density and morphology. Values were calculated for each fibre and averaged across fibres to create an average value for each participant. For mitochondrial area density, the summed mitochondrial area across the four fibres is presented as a percentage of the summed image area across the four fibres. The mitochondrial fragmentation index was calculated as the total mitochondrial number divided by the total mitochondrial area, and used as an indicator of the degree of mitochondrial fragmentation [4]. Morphological descriptors were also recorded, including: perimeter (µm), aspect ratio (i.e. the length-to-width ratio of a bounding box [major axis/minor axis]), maximal and minimal Ferret's diameter (i.e. the longest and shortest distances, respectively, between two parallel tangents at opposing boundaries of a given mitochondria, µm), the perimeter-to-area ratio (i.e. the 2-dimensional measure that is analogous to the surface area-to-volume ratio of a 3-dimensional object), circularity (i.e. 4π [area/perimeter^2], with a value of 1 indicating a perfect circle), and roundness (i.e. 4*area/[π*major axis^2]). Finally, the images used for the mitochondrial analysis (i.e., n = 926 in total) were scored on a scale of 0 (no lipid, little glycogen) to 5 (extreme lipid and glycogen accumulation) for glycogen and intramyocellular lipid deposition by three independent raters in a blinded and randomized fashion, as previously described [5]. Intramyocellular lipids were considered that had a minimum diameter of 200 nm and possessed a near spherical white-greyish appearance with a fuzzy border and absence of a distinguishable membrane [6].

Capillarization

Capillarization was assessed by staining for *Ulex Europaeus* Agglutin 1 lectin (UEA-1, B-1065-2, Vector Laboratories). Sections were air-dried for 10 minutes and then fixed in ice cold acetone (-20°C) for 15 minutes. Subsequently, slides were washed 3 times for 2 minutes in 1x PBS and blocked with 0.1% bovine serum albumin (SP-505 Vector Laboratories, Inc., Burlingame, CA, USA) for 30 minutes. Afterwards, slides were incubated with 20 µg/mL UEA-I (B-1065-2, Vector Laboratories) for 30 minutes at room temperature. Following this another washing step was performed, followed by incubation with VECTASTAIN ® Elite ABC-HRP Kit Peroxidase (PK-6100, Vector Laboratories) for 30 minutes at room temperature. Another washing step was performed, followed by incubation with ImmPACT™ AMEC Red Peroxidase Substrate (SK-4285, Vector Laboratories) for 10 minutes at room temperature. Finally, a washing step was performed in ddH_2O for 5 minutes and sections were mounted with glycerine-gelatin (pre-warmed to 37°C). Capillary density (the number of capillaries per mm^2) and the capillary-to-fibre ratio were calculated using ImageJ.

Succinate dehydrogenase activity

Succinate dehydrogenase (SDH) activity was determined using quantitative histochemistry. Immediately after sectioning, slides were air-dried for ten minutes, and incubated at 37°C in a medium containing 0.55 mM tetranitroblue tetrazolium (Sigma, St. Louis, USA), 0.2 M sodium succinate,14 mM sodium azide, and 0.1 M sodium phosphate buffer, pH 7.6. Biopsy sections were incubated for 20 minutes in the dark before the reaction was briefly stopped in 0.01 M HCl, washed and mounted with glycerine gelatin. Samples were subsequently stored at 4°C and images were made within 10 days of staining using a light microscope (Leica DMRB, Wetzlar, Germany) using a 10x objective, and absorbance was measured by microdensitometry with an interference filter at 660 nm using Image J (National Institutes of Health, Bethesda, USA). Weighted average SDH activity was determined from an average of 88 ± 22 individual fibres from each participant, with care taken to exclude fibres that were cut in a longitudinal orientation. Values were expressed as ΔA_{660} per µm tissue thickness per second of staining time ($\Delta A_{660} \cdot \mu m^{-1} \cdot s^{-1}$). For each participant, individual fibres were rank-ordered from highest to lowest for SDH activity. Subsequently, the 10 fibres with highest and lowest SDH activity for each subject were classified as high- and low-oxidative fibres, respectively.

Fiber-type composition

Skeletal muscle fiber type quantification and size were assessed using immunofluorescence techniques using primary antibodies against myosin heavy chain (MHC) I (BA-D5), MHC II (SC-71), and MHC IIx (6H1) [8]. A negative control, without primary antibody, was included for background correction. Firstly, sections were air-dried for 10 minutes and then blocked with 10% Normal Goat Serum (NGS) for 60 minutes. Subsequently, slides were washed 3 times for 5 minutes each in 1x phosphate-buffered saline (PBS) and incubated with the primary antibodies for 60 minutes at room temperature. Again, slides were washed and subsequently incubated with the secondary antibodies for 60 minutes in the dark at room temperature. Once more, a washing step was performed and subsequently incubation of the slides with Wheat Germ Agglutinin (WGA) for 30 minutes in the dark at room temperature, followed by a final washing step and mounting the sections with coverslips using Vectashield Vibrance. Analysis was performed with ImageJ and Sandia Matlab AnalysiS Hierarchy (SMASH) Toolbox (version 1.0) in Matlab (version 2022a).

Statistical analyses

All data are presented as group means ± SD unless otherwise indicated. Data normality was tested using a Shapiro-Wilkes normality test. Differences between group means were assessed using independent samples t-tests or Mann-Whitney U tests. Subgroup analysis was performed by splitting the type 1 diabetes group above and below the median of the following descriptive variables hypothesized to influence our outcome variables: age, disease duration, HbA1c, and body mass index (BMI). Subgroup analysis was then performed using one- or two-way ANOVA with Holm-Šídák post-hoc tests. Pearson’s correlation was used to explore relationships between variables of interest. All statistical analysis was performed using GraphPad Prism version 9 (GraphPad Software, La Jolla, CA) and significance was accepted when $P < 0.05$.

ESM Table 1. Cohort characteristics with type 1 diabetes group split by descriptive variables: HbA1c, BMI, $\dot{V}O_{2max}$, age, and short vs. long disease duration. Values displayed as mean ± SD.

	**Low HbA1c**	**High HbA1c**	**Low BMI**	**High BMI**	**Low $\dot{V}O_{2max}$**	**High $\dot{V}O_{2max}$**
n	7	9	8	8	8	8
Sex (male/female)	3/4	5/4	6/2	3/5	2/6	6/2
Age (years)	47 ± 14	36 ± 18	39 ± 17	43 ± 17	46 ± 18	35 ± 15
BMI (kg/m^2)	24 ± 2	25 ± 2	23 ± 1	26 ± 2**	26 ± 2	23 ± 2*
$\dot{V}O_{2max}$ (mL.kg^{-1}.min^{-1})	41 ± 10	39 ± 12	45 ± 9	34 ± 10*	31 ± 7	48 ± 6**
HbA_{1c} (mmol/mol)	39.0 ± 3.3	50.9 ± 4.4**	44.2 ± 8.7	47.5 ± 6.6	47.5 ± 6.6	44.2 ± 8.7
HbA_{1c} (%)	5.7 ± 0.3	6.9 ± 0.4**	6.2 ± 0.8	6.5 ± 0.6	6.5 ± 0.6	6.2 ± 0.8
Disease duration (yr)	22 ± 15	19 ± 18	19 ± 14	22 ± 19	27 ± 18	14 ± 12
	**Older**	**Younger**	**Short duration**	**Long duration**		
n	8/8	8/8	9	7		
Sex (male/female)	4/4	4/4	4/5	4/3		
Age (years)	56 ± 6	26 ± 7**	28 ± 9	57 ± 5**		
BMI (kg/m^2)	25 ± 2	24 ± 2	24 ± 2	25 ± 3		
$\dot{V}O_{2max}$ (mL.kg^{-1}.min^{-1})	43 ± 10	36 ± 11	43 ± 9	36 ± 12		
HbA_{1c} (mmol/mol)	43.2 ± 6.6	48.6 ± 8.7	48.6 ± 7.7	43.2 ± 6.6		
HbA_{1c} (%)	6.1 ± 0.6	6.6 ± 0.8	6.6 ± 0.7	6.1 ± 0.6		
Disease duration (yr)	34 ± 10	7 ± 7**	8 ± 8	36 ± 9**		

BMI, body mass index; $\dot{V}O_{2max}$, maximal oxygen uptake; $HbA1_c$; glycated haemoglobin. * indicates $P < 0.05$ compared with corresponding descriptive category group; ** indicates $P < 0.001$ compared with corresponding descriptive category group.

ESM Table 2. Cohort characteristics with type 1 diabetes group split by sex and maximal oxygen uptake. Values displayed as mean ± SD.

	**Controls (male)**	**Controls (female)**	**Type 1 diabetes (male)**	**Type 1 diabetes (female)**
n	8	7	8	8
Age (years)	36 ± 18	48 ± 13	39 ± 17	43 ± 18
BMI (kg/m^2)	24 ± 4	23 ± 3	23 ± 1	26 ± 2
$\dot{V}O_{2max}$ (mL.kg^{-1}.min^{-1})	45 ± 8	37 ± 5*	47 ± 8	33 ± 8*
HbA_{1c} (mmol/mol)	31.9 ± 2.9**	29.5 ± 3.8**	46.4 ± 8.7	45.3 ± 7.7
HbA_{1c} (%)	5.09 ± 0.27**	4.85 ± 0.35**	6.4 ± 0.8	6.3 ± 0.7
Disease duration (yr)	-	-	19 ± 14	22 ± 19

	**Controls (low $\dot{V}O_{2max}$)**	**Controls (high $\dot{V}O_{2max}$)**
n	7	8
Sex (male/female)	6/2	5/2
Age (years)	33 ± 15	52 ± 13
BMI (kg/m^2)	23 ± 3	25 ± 3
$\dot{V}O_{2max}$ (mL.kg^{-1}.min^{-1})	35 ± 3	46 ± 7$^{\#}$
HbA_{1c} (mmol/mol)	31.2 ± 2.7	31.2 ± 4.4
HbA_{1c} (%)	5.03 ± 0.25	5.03 ± 0.40
Disease duration (yr)	-	-

BMI, body mass index; $\dot{V}O_{2max}$, maximal oxygen uptake; $HbA1_c$; glycated haemoglobin. * indicates P < 0.05 compared with males of same disease status; ** indicates P < 0.001 compared with type 1 diabetes, # indicates P < 0.001 compared with low $\dot{V}O_{2max}$ controls.

ESM Figures

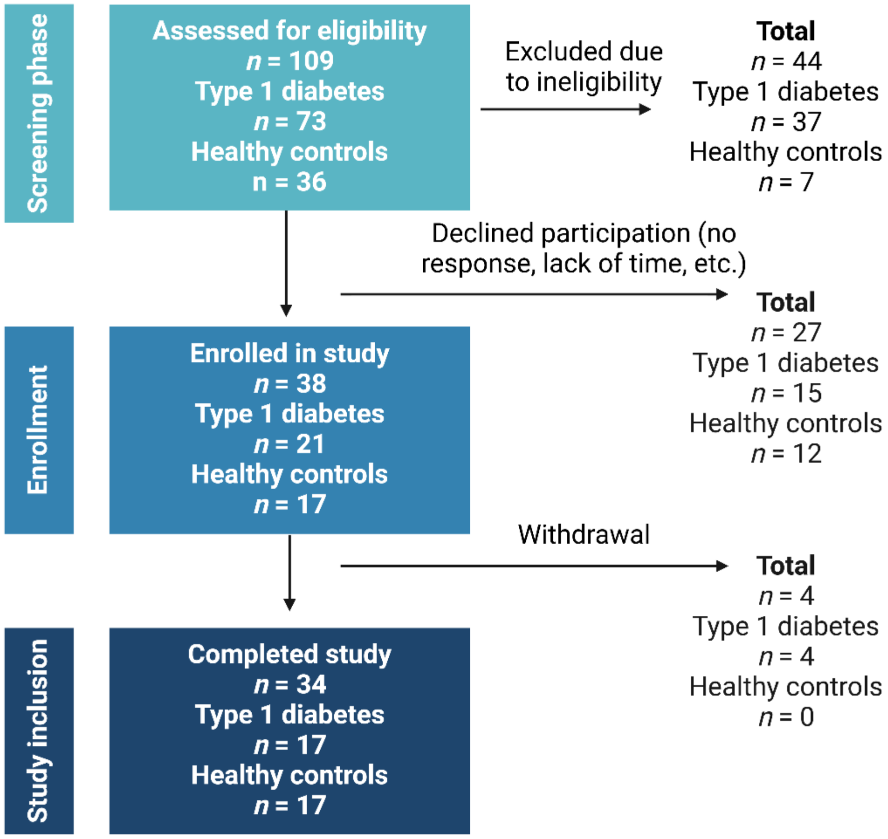

ESM Figure 1. Flowchart for participant screening process and recruitment. Out of 109 participants that initially expressed their interest in the study (n = 73 with type 1 diabetes and n = 36 healthy controls), 44 were immediately excluded for not meeting the eligibility criteria (n = 37 with type 1 diabetes and n = 7 healthy controls). A further 27 declined participation either due to a lack of further response or reasons such as lack of time (n = 15 with type 1 diabetes and n = 12 healthy controls). 38 individuals agreed to take part in the study (n = 21 with type 1 diabetes and n = 17 healthy controls). A further four individuals withdrew after agreeing to take part (all from the type 1 diabetes group) due to personal reasons. 34 individuals completed the study (n = 17 with type 1 diabetes and n = 17 healthy controls). Number of individuals included per analysis may vary due to tissue scarcity, the details are explained further in the text.

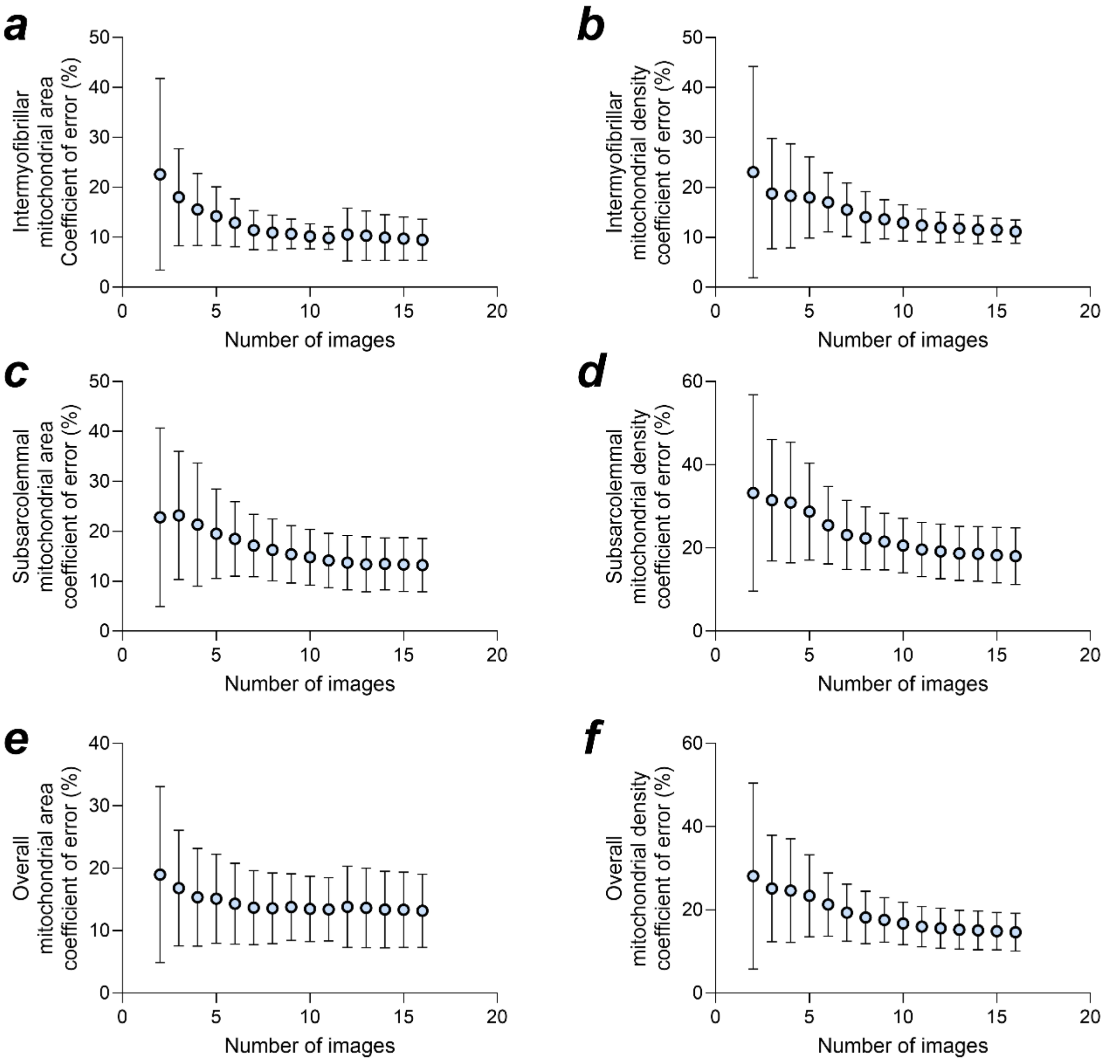

ESM Figure 2. Precision of mitochondrial transmission electron microscopy analyses. For each panel, the coefficient of error is plotted against the number of images analyzed. ***a***, intermyofibrillar mitochondrial area. ***b***, intermyofibrillar mitochondrial density. ***c***, subsarcolemmal mitochondrial area. ***d***, subsarcolemmal mitochondrial density. ***e***, overall mitochondrial area (mean of both regions). ***f***, overall mitochondrial density (mean of both regions). Circles represent group mean, error bars represent standard deviations (*n* = 30 participants).

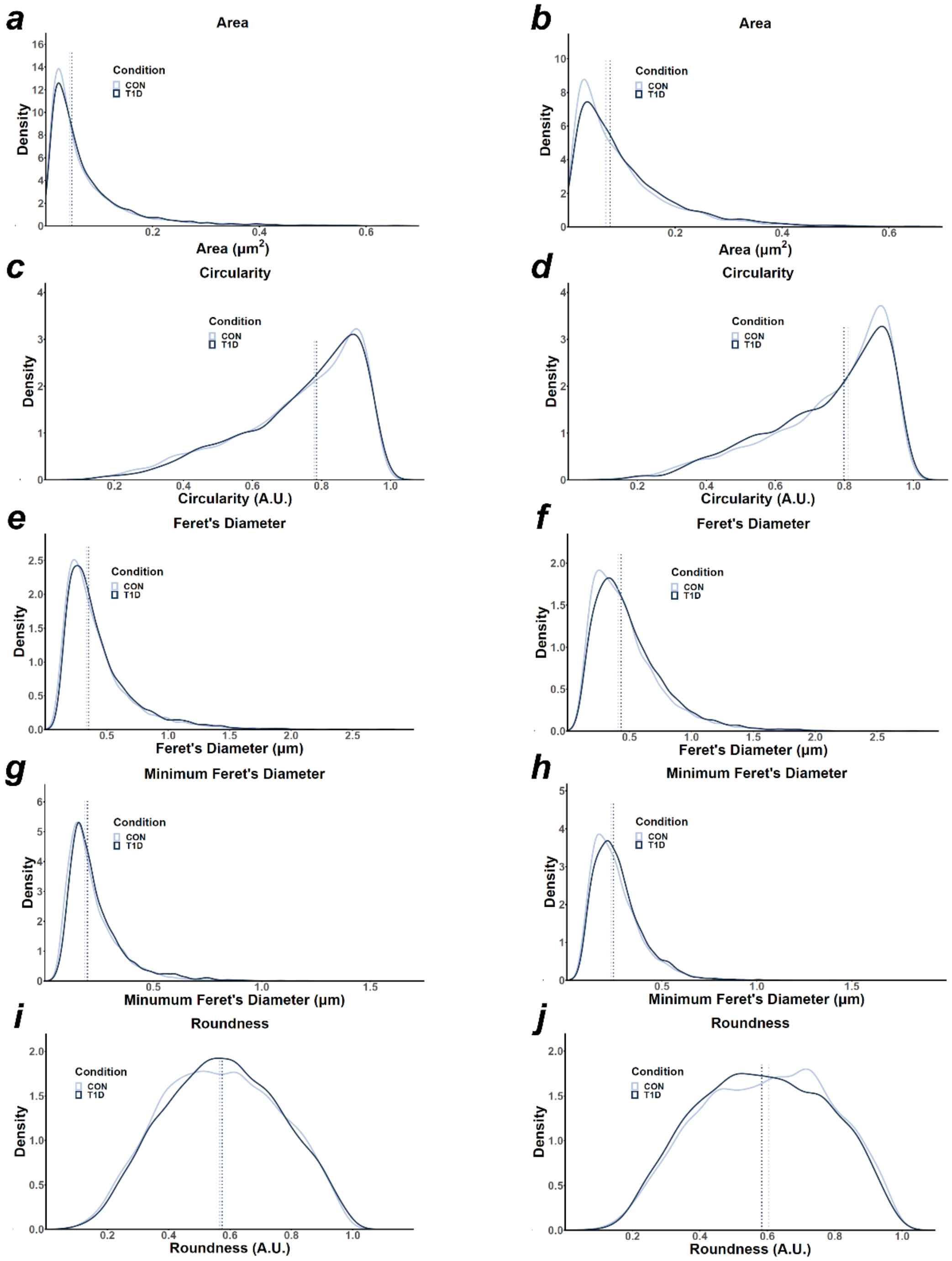

ESM Figure 3. Mitochondrial area and morphology distributions. Transmission electron microscopy was performed on single skeletal muscle fibres obtained via muscle biopsy. The morphology of muscle mitochondria was assessed by manually tracing the outlines of 23,200 mitochondrial profiles from 31 participants (*n* = 16 type 1 diabetes, *n* = 15 controls). Analyses were performed for both the intermyofibrillar (*left panels*, 13,345 mitochondrial profiles) and subsarcolemmal (*right panels*, 9,855 mitochondrial profiles) regions. Data are displayed for mitochondrial area (***a*** **&** ***b***), circularity (***c*** **&** ***d***), Feret's diameter (***e*** **&** ***f***), minimum Feret's diameter (***g*** **&** ***h***), and roundness (***i*** **&** ***j***). Density distributions are displayed as light (controls) and dark blue (type 1 diabetes) curves, with dashed lines indicating the median value for each distribution. No significant differences were observed.

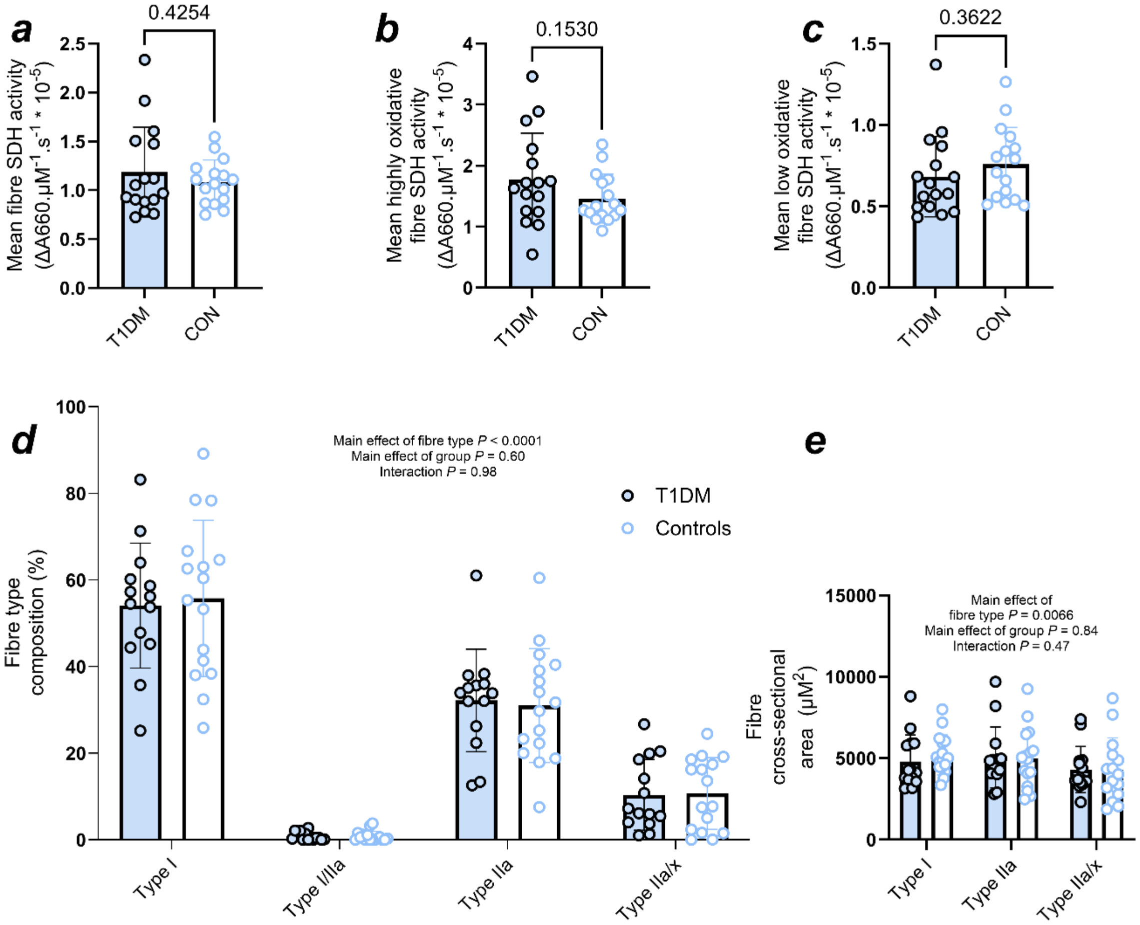

ESM Figure 4. (Immuno-)histochemical analyses of human biopsy material. Transverse muscle sections were stained for succinate dehydrogenase (SDH) activity (***a***-***c***) and myosin heavy chain I, IIa, and IIx for fibre type determination (***d***-***e***). ***a***, mean fibre SDH activity. ***b***, mean fibre SDH activity of the 10 fibres expressing the greatest SDH activity per participant (high-oxidative fibres). ***c***, mean fibre SDH activity of the 10 fibres expressing the lowest SDH activity per participant (low-oxidative fibres). ***d***, mean fibre type composition. ***e***, mean fibre cross-sectional area per fibre type. Individual *P* values for each comparison (main and interaction terms) displayed on each panel. CON, healthy controls; T1DM, type 1 diabetes mellitus.

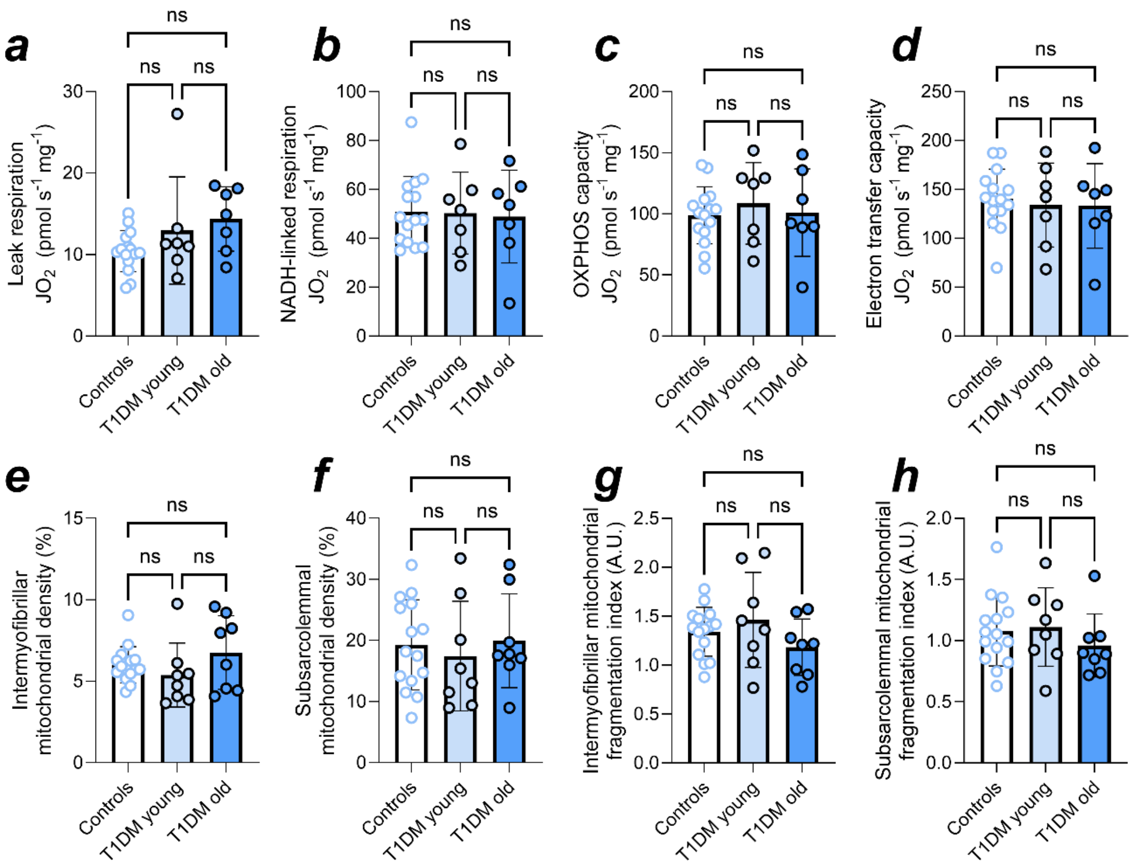

ESM Figure 5. Mitochondrial respiration, density and fragmentation in controls and younger (n = 8, 26 ± 7 years) and older (n = 8, 56 ± 6 years) individuals with type 1 diabetes. ***a***, leak respiration in the presence of NADH-linked substrates and no ADP. ***b***, NADH-linked respiration in the presence of NADH-linked substrates and saturating [ADP]. ***c***, maximal oxidative phosphorylation capacity in the presence of saturating [ADP] and convergent electron flow through complexes I and II. ***d***, electron transfer capacity measured in the presence of optimal concentrations of uncoupler. ***e***, intermyofibrillar mitochondrial density. ***f***, subsarcolemmal mitochondrial density. ***g***, intermyofibrillar mitochondrial fragmentation index. ***h***, Subsarcolemmal mitochondrial fragmentation index. ***a-d***, n=15 controls, n=7 younger type 1 diabetes, n=7 older type 1 diabetes. ***e-h***, n=15 controls, n=8 younger type 1 diabetes, n=8 older type 1 diabetes. ns, no significant difference. JO_2, mitochondrial oxygen consumption; T1DM, type 1 diabetes mellitus.

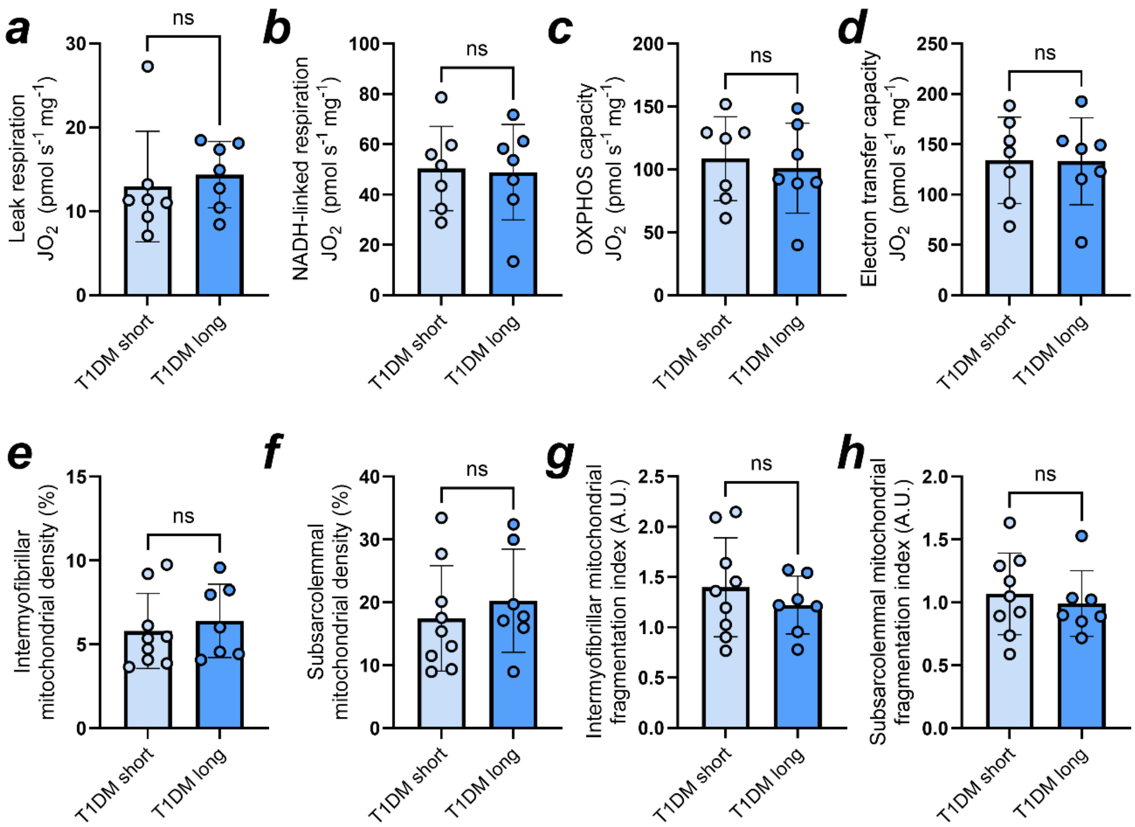

ESM Figure 6. Mitochondrial respiration, density and fragmentation in individuals with type 1 diabetes with longer (n = 7, 34 ± 10 years) and shorter (n = 9, 7 ± 7 years) disease duration. ***a***, leak respiration in the presence of NADH-linked substrates and no ADP. ***b***, NADH-linked respiration in the presence of NADH-linked substrates and saturating [ADP]. ***c***, maximal oxidative phosphorylation capacity in the presence of saturating [ADP] and convergent electron flow through complexes I and II. ***d***, electron transfer capacity measured in the presence of optimal concentrations of uncoupler. ***e***, intermyofibrillar mitochondrial density. ***f***, subsarcolemmal mitochondrial density. ***g***, intermyofibrillar mitochondrial fragmentation index. ***h***, Subsarcolemmal mitochondrial fragmentation index. ***a-d***, n=7 individuals with a shorter type 1 diabetes disease duration, n=7 individuals with a longer type 1 diabetes disease duration. ***e-h***, n=9 individuals with a shorter type 1 diabetes disease duration, n=7 individuals with a longer type 1 diabetes disease duration. ns, no significant difference. JO_2, mitochondrial oxygen consumption; T1DM, type 1 diabetes mellitus.

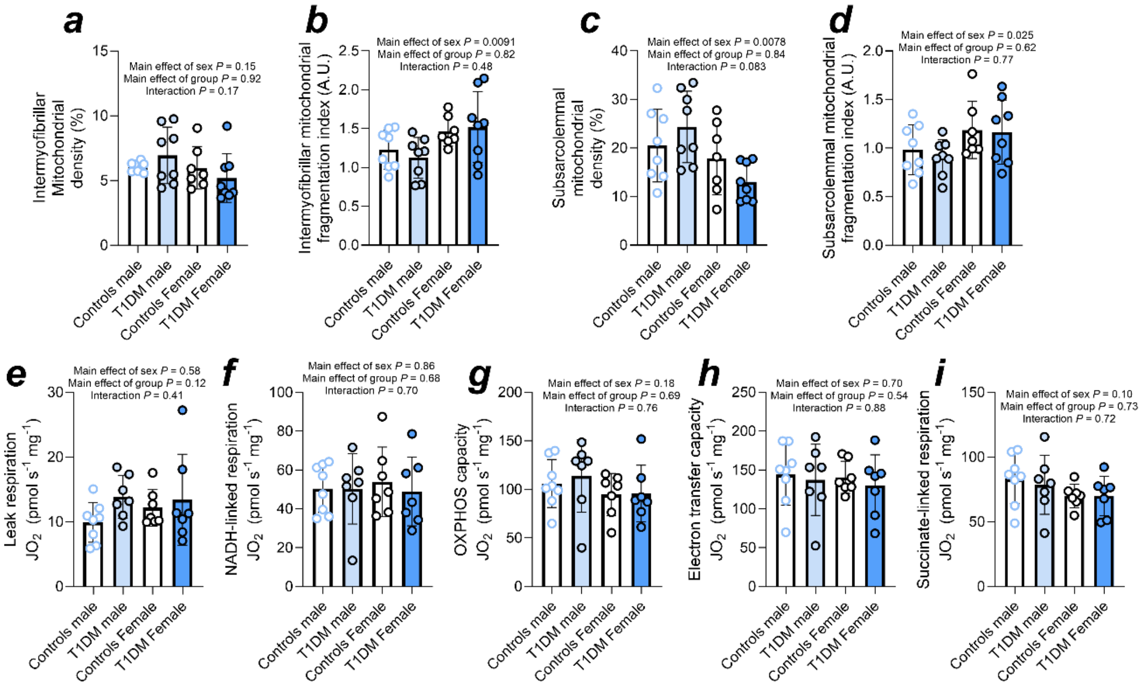

ESM Figure 7. Mitochondrial density, fragmentation, and respiration and healthy controls in individuals with type 1 diabetes grouped according to sex. ***a***, intermyofibrillar mitochondrial density. ***b***, intermyofibrillar mitochondrial fragmentation index. ***c***, subsarcolemmal mitochondrial density. ***d***, Subsarcolemmal mitochondrial fragmentation index. ***e***, leak respiration in the presence of NADH-linked substrates and no ADP. ***f***, NADH-linked respiration in the presence of NADH-linked substrates and saturating [ADP]. ***g***, maximal oxidative phosphorylation capacity in the presence of saturating [ADP] and convergent electron flow through complexes I and II. ***h***, electron transfer capacity measured in the presence of optimal concentrations of uncoupler. ***i***, Succinate-linked respiration measured in the presence of saturating ADP, NADH- and FADH-linked substrates, optimal concentrations of uncoupler and rotenone. ***a-d***, *n*=8 for males and females with type 1 diabetes, *n*=7 for female controls. ***e-i***, *n*=8 control males, *n*=7 males with type 1 diabetes, *n*=7 control females, *n*=7 males with type 1 diabetes. Individual *P* values for each statistical effect are displayed on each panel. No significant interaction effects were observed. JO_2, mitochondrial oxygen consumption; T1DM, type 1 diabetes mellitus.

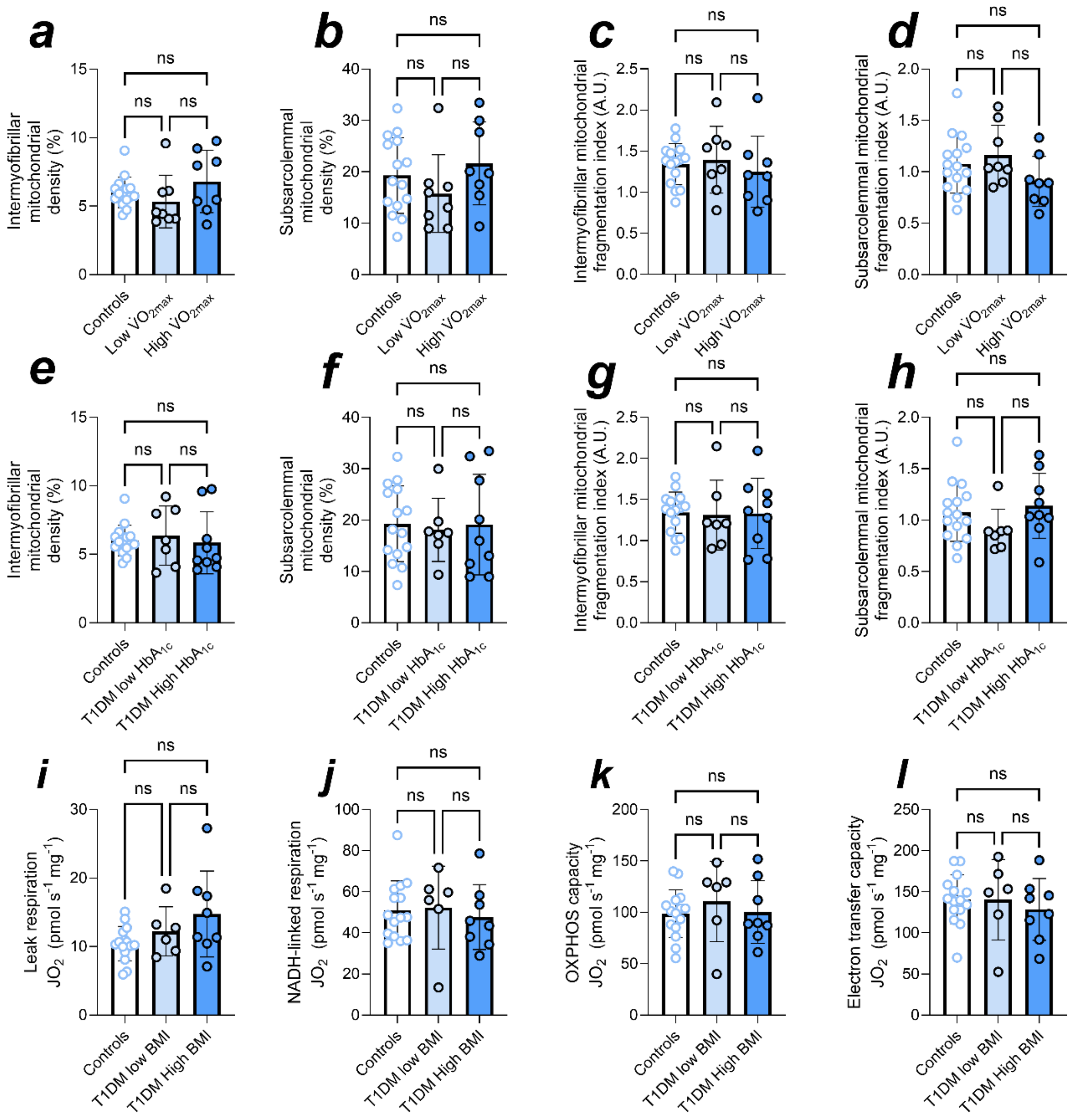

ESM Figure 8. Mitochondrial characteristics in healthy controls and individuals with type 1diabetes, with the type 1 diabetes group split by maximal oxygen uptake (***a-d***), glycated haemoglobin (HbA_{1c}) concentrations (***e-h***), and body mass index (BMI, ***i-l***). ***a*** & ***e***, intermyofibrillar mitochondrial density. ***b*** & ***f***, subsarcolemmal mitochondrial density. ***c*** & ***g***, intermyofibrillar mitochondrial fragmentation index. ***d*** & ***h***, Subsarcolemmal mitochondrial fragmentation index. ***i***, leak respiration in the presence of NADH-linked substrates and no ADP. ***j***, NADH-linked respiration in the presence of NADH-linked substrates and saturating [ADP]. ***k***, maximal oxidative phosphorylation capacity in the presence of saturating [ADP] and convergent electron flow through complexes I and II. ***l***, electron transfer capacity measured in the presence of optimal concentrations of uncoupler. ***a-d***, n=15 controls, n=8 type 1 diabetes low $\dot{V}O_{2max}$, n=8 type 1 diabetes high $\dot{V}O_{2max}$. ***e-h***, n=15 controls, n=7 type 1 diabetes low HbA_{1c}, n=9 type 1 diabetes high HbA_{1c}. ***i-l***, n=15 controls, n=6 type 1 diabetes low BMI, n=8 type 1 diabetes high BMI. ns, no significant difference. JO_2, mitochondrial oxygen consumption; T1DM, type 1 diabetes mellitus; OXPHOS, oxidative phosphorylation capacity.

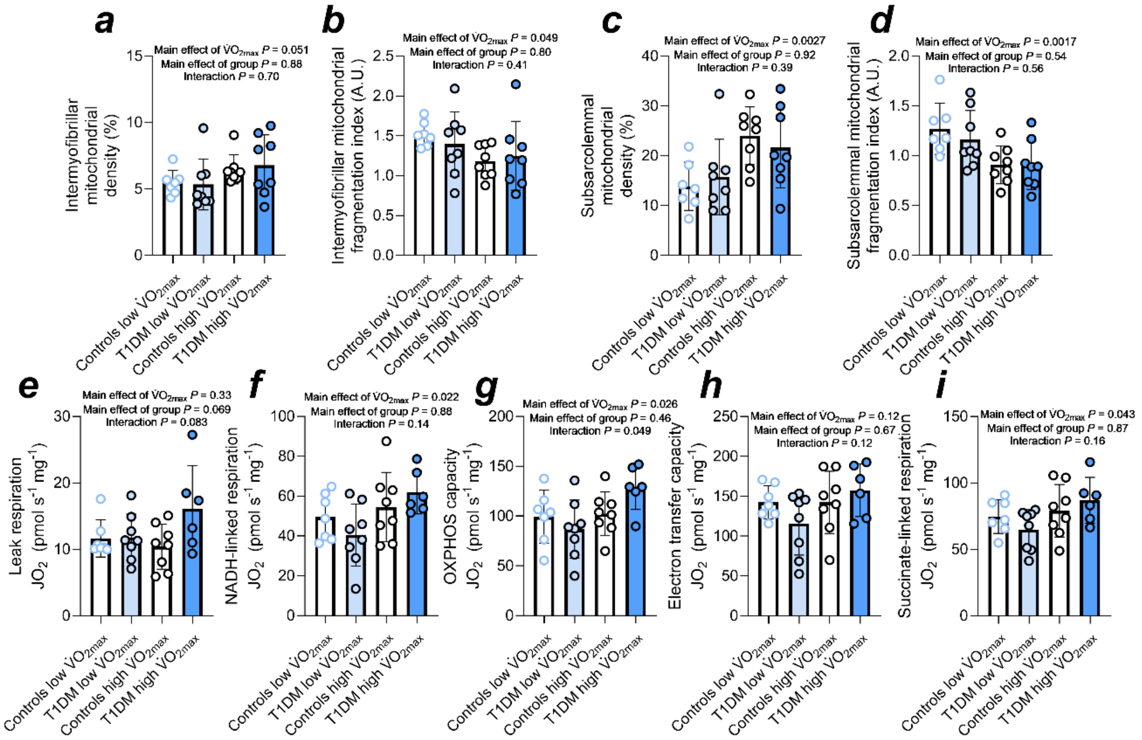

ESM Figure 9. Mitochondrial density, fragmentation, and respiration and healthy controls in individuals with type 1 diabetes grouped according to maximal oxygen uptake ($\dot{V}O_{2max}$). ***a***, intermyofibrillar mitochondrial density. ***b***, intermyofibrillar mitochondrial fragmentation index. ***c***, subsarcolemmal mitochondrial density. ***d***, Subsarcolemmal mitochondrial fragmentation index. ***e***, leak respiration in the presence of NADH-linked substrates and no ADP. ***f***, NADH-linked respiration in the presence of NADH-linked substrates and saturating [ADP]. ***g***, maximal oxidative phosphorylation capacity in the presence of saturating [ADP] and convergent electron flow through complexes I and II. ***h***, electron transfer capacity measured in the presence of optimal concentrations of uncoupler. ***i***, Succinate-linked respiration measured in the presence of saturating ADP, NADH- and FADH-linked substrates, optimal concentrations of uncoupler and rotenone. ***a-d***, *n*=8 for type 1 diabetes with high and low $\dot{V}O_{2max}$ and controls with high $\dot{V}O_{2max}$, *n*=7 for controls with low $\dot{V}O_{2max}$. ***e-i***, *n*=7 controls with low $\dot{V}O_{2max}$, *n*=8 controls with high $\dot{V}O_{2max}$, *n*=8 type 1 diabetes with low $\dot{V}O_{2max}$, *n*=6 type 1 diabetes with high $\dot{V}O_{2max}$. Individual *P* values for each statistical effect are displayed on each panel. Post-hoc tests for the significant interaction effect on OXPHOS capacity revealed no significant differences. JO_2, mitochondrial oxygen consumption; T1DM, type 1 diabetes mellitus; OXPHOS, oxidative phosphorylation capacity.

Supplemental references

1. Monaco CMF, Hughes MC, Ramos SV, et al (2018) Altered mitochondrial bioenergetics and ultrastructure in the skeletal muscle of young adults with type 1 diabetes. Diabetologia 61(6):1411–1423. https://doi.org/10.1007/s00125-018-4602-6

2. Nagi D, Gallen I (2010) ABCD position statement on physical activity and exercise in diabetes. Practical Diabetes International 27(4):158–163a. https://doi.org/10.1002/pdi.1471

3. Goulding RP, Roche DM, Scott SN, Koga S, Weston PJ, Marwood S (2020) Limitations to exercise tolerance in type 1 diabetes: the role of pulmonary oxygen uptake kinetics and priming exercise. Journal of Applied Physiology. https://doi.org/10.1152/japplphysiol.00892.2019

4. Gemmink A, Daemen S, Wefers J, et al (2023) Twenty-four hour rhythmicity in mitochondrial network connectivity and mitochondrial respiration; a study in human skeletal muscle biopsies of young lean and older individuals with obesity. Mol Metab 72:101727. https://doi.org/10.1016/j.molmet.2023.101727

5. Eggelbusch M, Charlton BT, Bosutti A, et al (2024) The impact of bed rest on human skeletal muscle metabolism. CR Med 5(1). https://doi.org/10.1016/j.xcrm.2023.101372

6. de Almeida ME, Ørtenblad N, Petersen MH, et al (2023) Acute exercise increases the contact between lipid droplets and mitochondria independently of obesity and type 2 diabetes. J Physiol 601(10):1797–1815. https://doi.org/10.1113/JP284386

7. Appelman B, Charlton BT, Goulding RP, et al (2024) Muscle abnormalities worsen after post-exertional malaise in long COVID. Nat Commun 15(1):17. https://doi.org/10.1038/s41467-023-44432-3

8. Horwath O, Envall H, Röja J, et al (2021) Variability in vastus lateralis fiber type distribution, fiber size, and myonuclear content along and between the legs. J Appl Physiol (1985) 131(1):158–173. https://doi.org/10.1152/japplphysiol.00053.2021
